# Inference of Network Dynamics and Metabolic Interactions in the Gut Microbiome

**DOI:** 10.1371/journal.pcbi.1004338

**Published:** 2015-06-23

**Authors:** Steven N. Steinway, Matthew B. Biggs, Thomas P. Loughran, Jason A. Papin, Reka Albert

**Affiliations:** 1 College of Medicine, Pennsylvania State University, Hershey, Pennsylvania, United States of America; 2 University of Virginia Cancer Center, University of Virginia, Charlottesville, Virginia, United States of America; 3 Department of Biomedical Engineering, University of Virginia, Charlottesville, Virginia, United States of America; 4 Department of Physics, Pennsylvania State University, University Park, Pennsylvania, United States of America; The Pennsylvania State University, UNITED STATES

## Abstract

We present a novel methodology to construct a Boolean dynamic model from time series metagenomic information and integrate this modeling with genome-scale metabolic network reconstructions to identify metabolic underpinnings for microbial interactions. We apply this in the context of a critical health issue: clindamycin antibiotic treatment and opportunistic *Clostridium difficile* infection. Our model recapitulates known dynamics of clindamycin antibiotic treatment and *C*. *difficile* infection and predicts therapeutic probiotic interventions to suppress *C*. *difficile* infection. Genome-scale metabolic network reconstructions reveal metabolic differences between community members and are used to explore the role of metabolism in the observed microbial interactions. *In vitro* experimental data validate a key result of our computational model, that *B*. *intestinihominis* can in fact slow *C*. *difficile* growth.

## Author Summary

The community of bacteria that live in our intestines (called the “gut microbiome”) is important to normal intestinal function, and destruction of this community has a causative role in diseases including obesity, diabetes, and even neurological disorders. *Clostridum difficile* is an opportunistic pathogenic bacterium that causes potentially life-threatening intestinal inflammation and diarrhea and frequently occurs after antibiotic treatment, which wipes out the normal intestinal bacterial community. We use a mathematical model to identify how the normal bacterial community interacts and how this community changes with antibiotic treatment and *C*. *difficile* infection. We use this model to identify bacteria that may inhibit *C*. *difficile* growth. Our model and subsequent experiments indicate that *Barnesiella intestinihominis* inhibits *C*. *difficile* growth. This result suggests that *B*. *intestinihominis* could potentially be used as a probiotic to treat or prevent *C*. *difficile* infection.

## Introduction

Human health is inseparably connected to the billions of microbes that live in and on us. Current research shows that our associations with microbes are, more often than not, *essential* for our health [1]. The microbes that live in and on us (collectively our “microbiome”) help us to digest our food, train our immune systems, and protect us from pathogens [2,3]. The gut microbiome is an enormous community, consisting of hundreds of species and trillions of individual interacting bacteria [4]. Microbial community composition often persists for years without significant change [5].

When change comes, however, it can have unpredictable and sometimes fatal consequences. Acute and recurring infections by *Clostridium difficile* have been strongly linked to changes in gut microbiota [6]. The generally accepted paradigm is that antibiotic treatment (or some other perturbation) significantly disrupts the microbial community structure in the gut, which creates a void that *C*. *difficile* will subsequently fill [7–10]. Such infections occur in roughly 600,000 people in the United States each year (this number is on the rise), with an associated mortality rate of 2.3% [11]. Each year, healthcare costs associated with *C*. *difficile* infection are in excess of $3.2 billion [11]. An altered gut flora has further been identified as a causal factor in obesity, diabetes, some cancers and behavioral disorders [12-17].

What promotes the stability of a microbial community, or causes its collapse, is poorly understood. Until we know what promotes stability, we cannot design targeted treatments that prevent microbiome disruption, nor can we rebuild a disrupted microbiome. Studying the system level properties and dynamics of a large community is impossible using traditional microbiology approaches. However, network science is an emerging field which provides a powerful framework for the study of complex systems like the gut microbiome [18–23]. Previous efforts to capture the essential dynamics of the gut have made heavy use of ordinary differential equation (ODE) models [24,25]. Such models require the estimation of many parameters. With so many degrees of freedom, it is possible to overfit the underlying data, and it is difficult to scale up to larger communities [26,27]. Boolean dynamic models, conversely, require far less parameterization. Such models capture the essential dynamics of a system, and scale to larger systems. Boolean models have been successfully applied at the molecular [28,29], cellular [20], and community levels [30]. Here we present the first Boolean dynamic model constructed from metagenomic sequence information and the first application of Boolean modeling to microbial community analysis.

We analyze the dynamic nature of the gut microbiome, focusing on the effect of clindamycin antibiotic treatment and *C*. *difficile* infection on gut microbial community structure. We generate a microbial interaction network and dynamical model based on time-series data from metagenome data from a population of mice. We present the results of a dynamic network analysis, including steady-state conditions, how those steady states are reached and maintained, how they relate to the health or disease status of the mice, and how targeted changes in the network can transition the community from a disease state to a healthy state. Furthermore, knowing *how* microbes positively or negatively impact each other—particularly for key microbes in the community—increases the therapeutic utility of the inferred interaction network. We produced genome-scale metabolic reconstructions of the taxa represented in this community [31], and probe how metabolism could—and could not—contribute to the mechanistic underpinnings of the observed interactions. We present validating experimental evidence consistent with our computational results, indicating that a member of the normal gut flora, *Barnesiella*, can in fact slow *C*. *difficile* growth.

## Methods

### Data Sources

**Fig 1:**
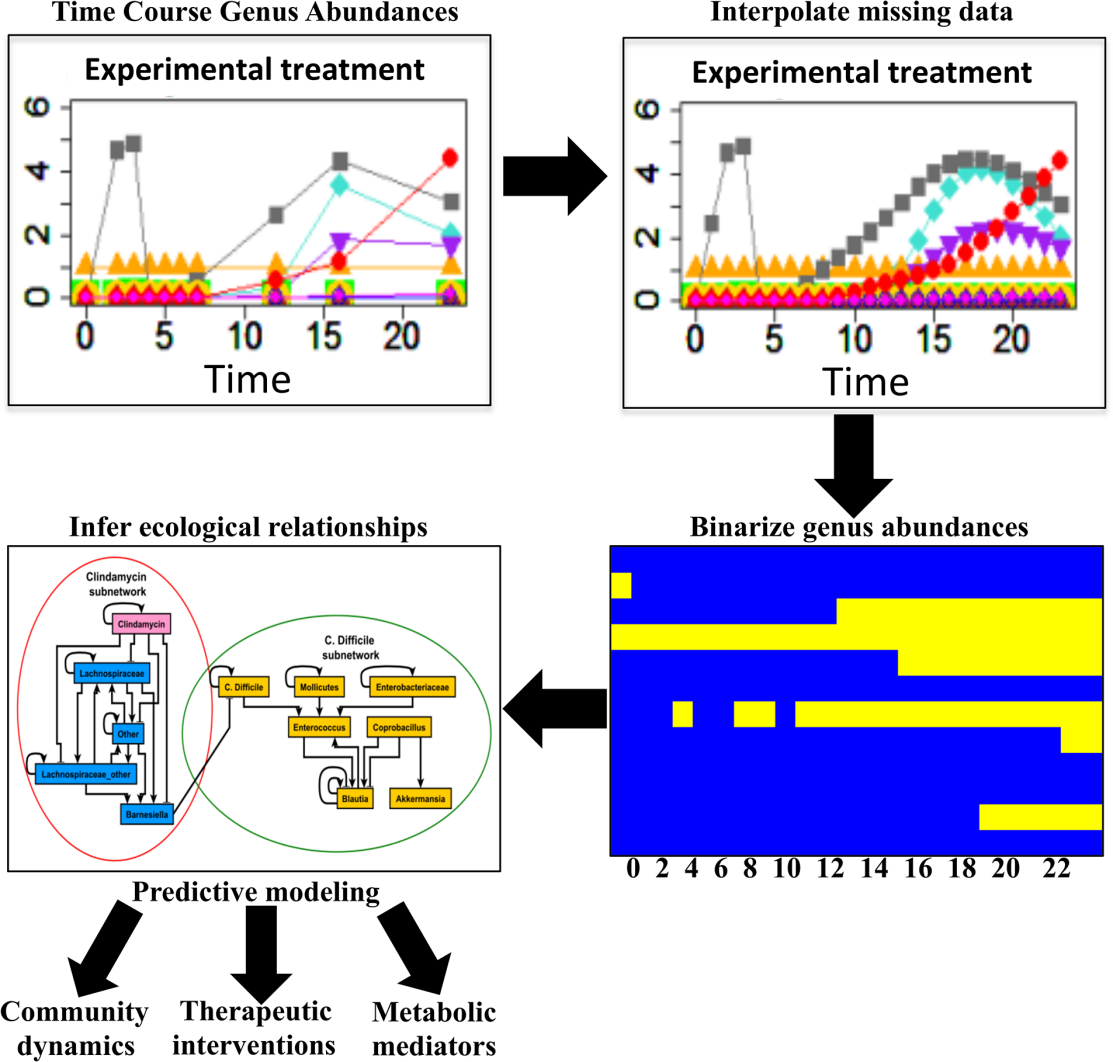
Dynamic analysis workflow. Time course genus abundance information was acquired from metagenomic sequencing of mouse gastrointestinal tracts under varying experimental conditions. Missing time points from experimental data were estimated such that genus abundances existed at the same time points across all treatment groups. Next, genus abundances were binarized such that Boolean regulatory relationships could be inferred. A dynamic Boolean model was constructed to explore gut microbial dynamics, therapeutic interventions, and metabolic mediators of bacterial regulatory relationships.

Buffie *et al*. reported treating mice with clindamycin and tracking microbial abundance by 16S sequencing [32]. Mice treated with clindamycin were more susceptible to *C*. *difficile* infection than controls. The collection of 16S sequences corresponding to these experiments was analyzed by Stein *et al*. [24]. First, Stein *et al*. aggregated the data by quantifying microbial abundance at the genus level. Abundances of the ten most abundant genera and an “other” group were presented as operational taxonomic unit (OTU) counts per sample. We use the aggregated abundances from Stein *et al*. as the starting point for our modeling pipeline (Fig 1).

This processed dataset consisted of nine samples and three treatment groups (n = 3 replicates per treatment group). The first treatment group (here called “Healthy”) received spores of *C*. *difficile* at t = 0 days, and was used to determine the susceptibility of the native microbiota to invasion. The second treatment group (here called “clindamycin treated”) received a single dose of clindamycin at t = -1 days to assess the effect of the antibiotic alone, and the third treatment group (here called “clindamycin+ *C*. *difficile* treated”) received a single dose of clindamycin (at t = -1 days) and, on the following day, was inoculated with *C*. *difficile* spores (S1A Fig). Under the clindamycin+ *C*. *difficile* treatment group conditions, *C*. *difficile* could colonize the mice and produce colitis; however this was not possible under the first two treatment group conditions.

### Interpolation of Missing Genus Abundance Information

**Fig 2:**
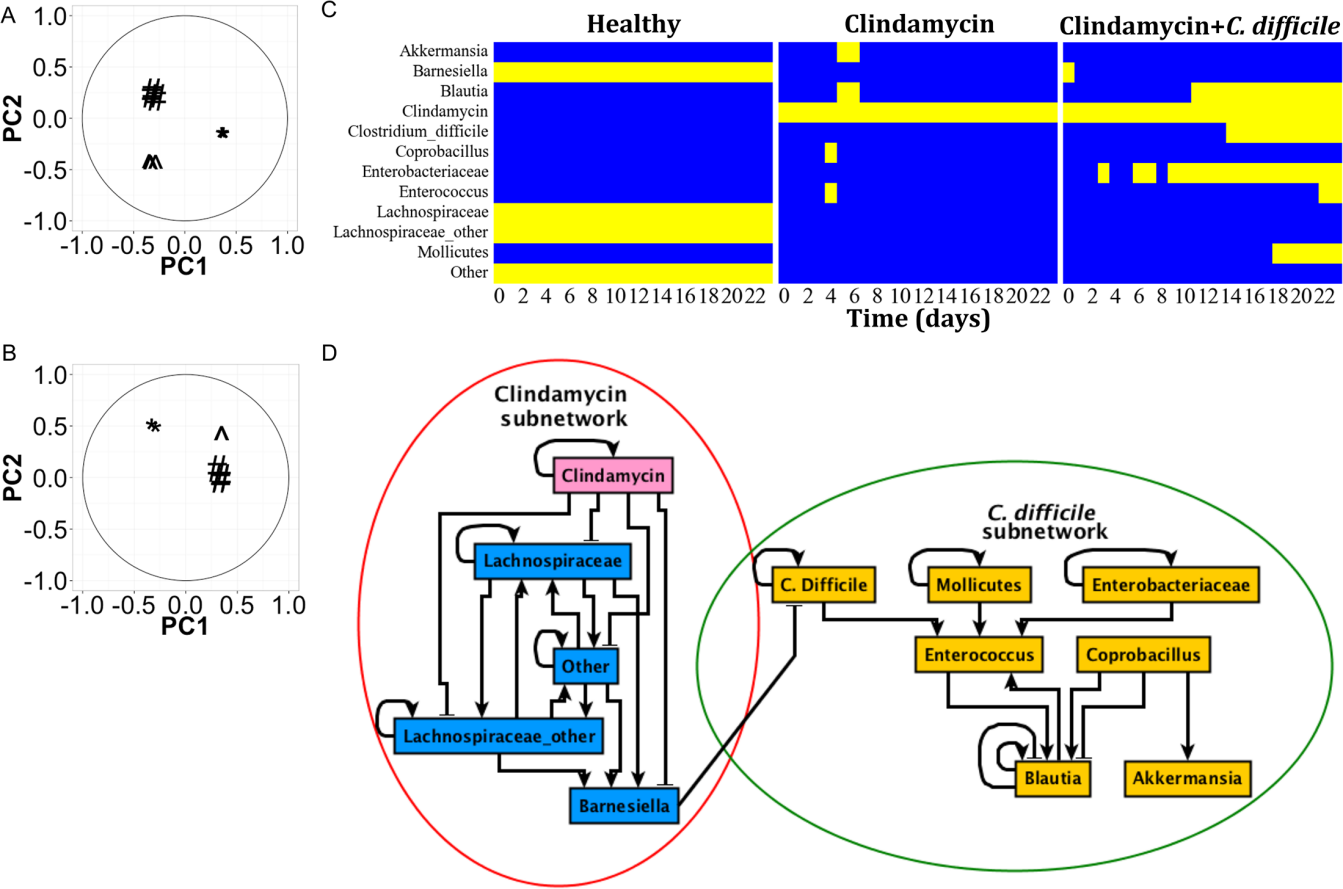
Construction of a network model of the gut microbiome from time course metagenomic genus abundance information. Principal component analysis coefficients associated with each sample in the metagenomic genus abundance dataset was completed for A) interpolated genus abundances and B) binarized interpolated genus abundances. ‘*’ = Healthy; ‘^’ = clindamycin treated; ‘#’ = clindamycin+ C. difficile treated. C) Consensus binarization of genus abundance information. Each heatmap represents the consensus binarization for each treatment group. The horizontal axis represents the day of the experiment that the sample came from. The vertical axis represents the specific genera being modeled. Each genus was binarized to a 1 (ON; above activity threshold) or 0 (OFF; below activity threshold). D) Interaction rules were inferred from the binarized data. The interaction rules were simplified for visualization (compound rules were broken into simple one-to-one edges).

The gut bacterial genus abundance dataset included some variation in terms of time points in which genera were sampled. That is, genus abundances were measured between 0 to 23 days; however, not all samples had measurements at all the time points (S1A Fig). Particularly, the healthy population only included time points at 0, 2, 6, and 13 days and Sample 1 of clindamycin+ *C*. *difficile* treated population was missing the 9 day time point. Missing abundance values for these 4 points were estimated using an interpolation approach (S1B Fig). For healthy samples, the 16 and 23 day time points could not be interpolated as the last experimentally identified time point for these samples is at 13 days. The assumption of the approximated polynomial for these samples is that extrapolated data points are linear using the slope of the interpolating curve at the nearest data point. Because genera abundances are fairly stable across time in this treatment group (i.e. the slope of most of the genera abundances is approximately zero), extrapolating two time points was deemed reasonable. A principal component analysis was completed on the interpolated data (Fig 2A) and shows that the interpolated time series bacterial genus abundance data clusters by experimental treatment group in the first two principal components. Furthermore, the results of the binarization for the healthy population suggest that interpolation did not have any concerning effects on the 16 and 23 day time points (S2 Fig).

Natural cubic spline interpolation was used to estimate genus abundances at missing time points in some samples. A cubic spline is constructed of piecewise third order (cubic) polynomials which pass through the known data points and has continuous first and second derivatives across all points in the dataset. Natural cubic spline is a cubic spline that has a second derivative equal to zero at the end points of the dataset [33]. Natural splines were interpolated such that all datasets had time points at single day intervals through the 23 day time point (S1B Fig).

### Network Modeling Framework

We use a Boolean framework in which each network node is described by one of two qualitative states: ON or OFF. We chose this framework because of its computational feasibility and capacity to be constructed with minimal and qualitative biological data [34]. The ON (logical 1) state means an above threshold abundance of a bacterial genus whereas the OFF (logical 0) state means below-threshold genus absence. The putative biological relationships among genera are expressed as mathematical equations using Boolean operators [29,34]. We inferred putative Boolean regulatory functions for each node, which are able to best capture the trends in the bacterial abundances. These rules, (edges in the interaction network) can be assigned a direction, representing information flow, i.e. effect from the source (upstream) node to the target (downstream) node. Furthermore, edges can be characterized as positive (growth promoting) or negative (growth suppressing). An additional layer of network analysis is the dynamic model, which is used to express the behavior of a system over time by characterizing each node by a state variable (e.g., abundance) and a function that describes its regulation. Dynamic models can be categorized as continuous or discrete, according to the type of node state variable used. Continuous models use a set of differential equations; however, the paucity of known kinetic details for inter-genus and/or inter-species interactions makes these models difficult to implement.

### Binarization

Genus abundance data was binarized (converted to a presence-absence dataset) to enable inference of Boolean relationships for modeling applications. We adapted a previously developed approach called iterative k-means binarization with a clustering depth of 3 (KM3) for this purpose [35]. This approach was employed because binarized data is able to maintain complex oscillatory behavior in Boolean models constructed from this data, whereas other binarization approaches fail to maintain these features [35].

Briefly, this approach uses k-means clustering with a depth of clustering *d* and an initial number of clusters *k* = 2^*d*^. In each iteration, data for a specific genus *G* are clustered into k unique clusters *C* ^1^_*G*_,…,*C* ^*k*^_*G*_, then for each cluster, *C* ^*n*^_*G*_, all the values are replaced by the mean value of *C* ^*n*^_*G*_. For the next iteration, the value of *d* is decreased and clustering is repeated. This methodology is repeated until *d* = 1. This approach, with *d* = 3 (called here as KM3 binarization) has previously been demonstrated as a superior binarization methodology to other binarization approaches for Boolean model construction because it conserves oscillatory behavior [35]. These analyses were performed using custom Python code based on a previously written algorithm [35] and is available in the supplemental materials.

Because KM3 binarization has a stochastic component (the initial grouping of binarization clusters), we employed KM3 binarization on the entire bacterial genus abundance time series dataset 1000 times. The average binarization for each sample (S2 Fig) was used to determine the most probable binarized state of each genus in each sample at each time point (S3 Fig). A principal component analysis of the most probable binarized genus abundances for each sample demonstrates that as with the continuous time series abundances (Fig 2A), binarized bacterial genus abundance data cluster by experimental treatment group (Fig 2B). For inference of Boolean rules from the binarized genus abundances (S3 Fig), the consensus of two of three samples for each treatment population was used as the binarized state of each genus at each time point in each sample (Fig 2C).

### Inference of Boolean Rules from Time Series Genus Abundance Information

The Best-fit extension was applied to learn Boolean rules from the binarized time series genus abundance information [36]. For each variable (genus) *X*_*i*_ in the binarized time series genus abundance data, Best-fit identifies the set of Boolean rules with k variables (regulators) that explains the variable’s time pattern with the least error size. The algorithm uses partially defined Boolean functions *pdBf* (*T*, *F*), where the set of true (T) and false vectors (F) are defined as *T* = {*X*′ ∈ {0, 1}^*k*^: *X*_*i*_ (*t* + 1) = 1} and *F* = {*X*′ ∈ {0, 1}^*k*^: *X*_*i*_ (*t* + 1) = 0}. Intuitively, the partial Boolean function summarizes the states of the putative regulators that correspond to a turning ON (T) or turning OFF (F) of the target variable. The error size *ε* of *pdBf*(T,F) is defined as the minimum number of inconsistencies within *X*′ that best classifies the T and F values of the dataset. The Best-Fit extension works by identifying smallest size *X*′ for *X*_*i*_. For more detailed information refer to [36]. In line with this, we considered the most parsimonious representation of the rules with the smallest *ε*. If the most parsimonious rule was self-regulation, we also considered rules with the same *ε* that included another regulator. If multiple rules fit these criteria for a given *X*_*i*_, it implied that they can independently represent the inferred regulatory relationships. In cases where the alternatives had the same value of (non-zero) *ε*, we explored combinations (such as appending them by an OR rule) and used the combination that best described the experimentally observed final (steady state) outcomes. For example, we combined the two alternative rules for *Blautia* with an OR relationship. In the case of *Barnesiella*, we chained three rules ("Other", "Lachnospiraceae_other", "Lachnospiraceae") by an OR relationship, and "not Clindamycin" by an AND relationship to incorporate the loss of *Barnesiella* in the presence of clindamycin (Fig 2C). This was also done for rules for “Lachnospiraceae”, “Lachnospiraceae_other” and “Other” and all four nodes attained the same rule. There are six nodes with multiple inferred (alternative) rules: “Barnesiella”,”Blautia”,”Enterococcus”,”Lachnospiraceae”,”Lachnospiraceae_other”, and”Other” had 4, 2, 5, 4, 4, and 4 rules, respectively. The six other nodes had a single inferred rule. The network in Fig 2C represents the union of all of the alternative rules produced by Best-Fit, or in other words,–it is a super-network of all alternative rules. Any alternative networks would be a sub-network of what we show. A strongly connected component between the nodes inhibited by clindamycin is a feature of the vast majority of these sub-networks. We used the implementation of Best-Fit in the R package BoolNet [37].

### Dynamic Analysis

Dynamic analysis is performed by applying the inferred Boolean functions in succession until a steady state is reached. Boolean models and discrete dynamic models in general focus on state transitions instead of following the system in continuous time. Thus, time is an implicit variable in these models. The network transitions from an initial condition (initial state of the bacterial community) until an attractor is reached. An attractor can be a fixed point (steady state) or a set of states that repeat indefinitely (a complex attractor). The basin of attraction refers to the set of initial conditions that lead the system to a specific attractor. For the network under consideration, the complete state space can be traversed by enumerating every possible combination of node states (**2** ^**12**^) and applying the inferred Boolean functions (or “update rules”) to determine paths linking those states. The state transition network describes all possible community trajectories from initial conditions to steady states, given the observed interactions between bacteria in the community.

We made use of two update schemes to simulate network dynamics: synchronous (deterministic) and asynchronous (stochastic). Synchronous models are the simplest update method: all nodes are updated at multiples of a common time step based on the previous state of the system. The synchronous model is deterministic in that the sequence of state transitions is definite for identical initial conditions of a model. In asynchronous models, the nodes are updated individually, depending on the timing information, or lack thereof, of individual biological events. In the general asynchronous model used here, a single node is randomly updated at each time step [38]. The general asynchronous model is useful when there is heterogeneity in the timing of network events but when the specific timing is unknown. Due to the heterogeneous mechanisms by which bacteria interact, we made the assumption of time heterogeneity without specifically known time relationships. Synchronous and asynchronous Boolean models have the same fixed points, because fixed points are independent of the implementation of time. However, the basin of attraction of each fixed point (i.e. the initial conditions that lead to each fixed point) may differ between synchronous and asynchronous models (S2 Table). For identification of all of the fixed points in the network (the attractor landscape), the synchronous updating scheme was used. However, for the perturbation analysis, the asynchronous updating scheme was used because it more realistically models the possible trajectories in a stochastic and/or time-heterogeneous system. The simulations of the gut microbiome model were performed using custom Python code built on top of the BooleanNet Python library, which facilitates Boolean simulations [39]. Our custom Python code is available in the supplemental materials.

### Perturbation Analysis

To capture the effect of removal (knockout) or addition (probiotic; forced over abundance) of genera, modification of the states/rules to describe removal or addition states were performed. These modifications were implemented in BooleanNet by setting the corresponding nodes to either OFF (removal) or ON (addition) and then removing the corresponding updating rules for these nodes for the simulations. By examining many such forced perturbations, we can identify potential therapeutic strategies, many of which may not be obvious or intuitive, particularly as network complexity increases. We used asynchronous update when simulating the effect of perturbations on the microbial communities. In each case we performed 1000 simulations and report the percentage of simulations that achieve a certain outcome.

### Generating Genus-Level Genome-Scale Metabolic Reconstructions

To generate draft metabolic network reconstructions for each of the ten genera in the paper, we first obtained genome sequences for representative species by searching the “Genomes” database of the National Center for Biotechnology Information (NCBI). Complete genomes for the first ten (or if less than ten, all) species within the appropriate genus were downloaded. During the process of reconstructing genus-level metabolic reconstructions, some genera were underrepresented (fewer than 10 species genomes) in the NCBI Genome database, including *Akkermansia*, *Barnesiella* and *Coprobacillus* (S3 Table). The search result order is based on record update time, and so it is quasi-random. Genomes were uploaded to the rapid annotations using subsystems technology (RAST) server for annotation [40]. Draft metabolic network reconstructions were generated by providing the RAST annotations to the Model SEED service [41]. Metabolic network reconstructions were downloaded in “.xls” format. Genus-level metabolic reconstructions were produced by taking the union of all species-level reconstructions corresponding to each genus, as has been done previously [42]. The one exception was *C*. *difficile*, which was produced by taking the union of three strain-level reconstructions.

### Subsystem Enrichment Analysis

Subsystems were defined as the Kyoto Encyclopedia of Genes and Genomes (KEGG) map with which each reaction was associated [43,44]. These associations were determined based on annotations in the Model SEED database [41]. To quantify enrichment, the complete set of unique reactions from all genus-level reconstructions was pooled, and the subsystem annotations corresponding to those reactions were counted. To determine enrichment for a given subset of the community (either a single genus-level reconstruction, or a set of reconstructions corresponding to a subnetwork), the subsystem occurrences were counted within the subset. The probability of a reconstruction containing *N* total subsystem annotations, with *M* or more occurrences of subsystem *I*, was determined by taking the sum of a hypergeometric probability distribution function (PDF) from *M* to the total occurrences of subsystem *I* in the overall population. Enrichment analysis was performed in Matlab [45].

### Identifying Seed Sets and Defining Metabolic Competition and Mutualism Scores

To quantify metabolic interactions, we started by utilizing the seed set detection algorithm developed by Borenstein *et al*. [46,47]. The algorithm follows three steps:

1. The genome-scale metabolic network reconstruction is reduced into simple one-to-one edges, such that for each reaction, each substrate and product pair forms an edge (e.g. *A* + *B* → *C* would become *A* → *C* and *B* → *C*).
2. The network is divided into strongly connected components, those groups of nodes for which two paths of opposite directions (e.g. *A* → *B* and *B* → *A*) exist between any two nodes in the group.
3. Nodes (and strongly connected components with five or fewer nodes) for which there are exclusively outgoing edges are defined as “inputs” to the model, or seed metabolites.

The rationale is that metabolites that feed into the network, but cannot be produced by any reactions within the network, must be obtained from the environment.

Competition metrics were generated following the process of Levy and Borenstein [46]. For a given pair of genera, the competition score is defined as:

$$CompScore_{ij}=\frac{\left|SeedSet_{i}\cap SeedSet_{j}\right|}{\left|SeedSet_{i}\right|}$$ (1)

Here *SeedSet*_*i*_ is the set of obligatory input metabolites to the metabolic network reconstruction for genus *i*, and |*SeedSet*_*i*_| is the number of metabolites contained in *SeedSet*_*i*_. The competition score indicates the fractional overlap of inputs that genus *i* shares with genus *j*, and so ranges between zero and one. The higher the score, the more similar the metabolic inputs to the two networks, making competition more likely.

For a given pair of genera, the mutualism score is defined as:

$$MutualismScore_{ij}=\frac{\left|SeedSet_{i}\;\cap \neg SeedSet_{j}\right|}{\left|SeedSet_{i}\right|}$$ (2)

Here ¬*SeedSet*_*j*_ is the set of metabolites that can be produced by the metabolic network for species *j* (i.e. all non-seed metabolites). The mutualism score indicates the fractional overlap of inputs that genus *i* consumes which genus *j* can potentially provide. The mutualism score ranges between zero and one. The higher the score, the more potential there is for nutrient sharing between species. While the score does not measure “mutualism” per se (it cannot necessarily distinguish between other interactions such as commensalism or amenalism [48]), for simplicity, we will refer to these scores as the competition and mutualism scores.

All metabolic reconstructions, seed sets, competition scores and mutualism scores are available in the supplemental materials. Seed set generation was performed using custom Matlab scripts, which are available in the supplement. [45]. Statistical tests were performed in R [49].

### Co-culture and Spent Media Experiments

*Barnesiella intestinihominis* DSM 21032 and *Clostridium difficile* VPI 10463 were grown anaerobically in PRAS chopped meat medium (CMB) (Anaerobe Systems, Morgan Hill, CA) at 37 C. To prepare *B*. *intestinihominis* spent medium, *B*. *intestinihominis* was grown in CMB until stationary phase (44 hours). The saturated culture was centrifuged, and the supernatant was filter sterilized (0.22 μM pore size). Growth curves were obtained by inoculating batch cultures in 96-well plates and gathering optical density measurements (870 nm) using a small plate reader that fits in the anaerobic chamber [50]. Single cultures were inoculated from overnight liquid culture to a starting density of 0.01. The co-cultures were started at a 1:1 ratio, for a total starting density of 0.02. Optical density was measured every 2 minutes for 24 hours, and the resulting growth curves were analyzed in Matlab [45]. Maximum growth rates were calculated by fitting a smooth line to each growth curve, and finding the maximum growth rate from among the instantaneous growth rates over the whole time course: [log(OD_t+1_)—log(OD_t_)] / [t_+1_-t]. The achieved bacterial density—area under the growth curve (AUC)—in a culture was calculated by integrating over the growth curve in each experiment using the “trapz()” function in Matlab. It can be thought of as representing the total biomass produced over time. The simply additive null model was calculated by fitting a Lotka-Volterra model [24] to the single cultures for both *B*. *intestihominis* and *C*. *difficile*. The null model of co-culture (assuming zero interaction between species) was simulated by using the parameters from single culture, and summing the predicted OD870 values.

All scripts used to analyze the data are available at https://bitbucket.org/gutmicrobiomepaper/microbiomenetworkmodelpaper/wiki/Home.

## Results

### Processing of a Microbial Genus Abundance Dataset for Network Inference

To capture the dynamics of inter-genus interactions in the intestinal tract we employed a pipeline (Fig 1) which translates metagenomic genus abundance information into a dynamic Boolean model. This approach involves three steps: 1) discretization (binarization) of genus abundances, 2) learning Boolean relationships among genera, and 3) translation of genus associations into a Boolean (discrete) dynamic model.

### Construction of a Dynamic Network Model from Binarized Time Series Microbial Genus Abundance Information

Boolean rules (S1 Table) were inferred from the time series binarized genus abundances using an implementation of the Best-fit extension [36] in the R Boolean network inference package BoolNet [37](see Methods). A network of 12 nodes and 33 edges was inferred (Fig 2D). The inferred interaction network has a clustered structure: the cluster (subnetwork) containing the two *Lachnospiraceae* nodes and *Barnesiella* is strongly influenced by clindamycin whereas the other subnetwork is largely independent of the first, except for the single edge between *Barnesiella* and *C*. *difficile* (Fig 2D). In fact, *Lachnospiraceae* nodes, *Barnesiella* and the group of “Other” genera form a strongly connected component; that is, every node is reachable from every other node. Most nodes of the second subnetwork are positively influenced by *C*. *difficile*, with the exception of *Coprobacillus*, for which no regulation by other nodes was inferred, and *Akkermansia*, which is inferred to be regulated only by *Coprobacillus*. These latter two genera are transiently present (around day 5) in the clindamycin treatment group, but they do not appear in the final states of any of the treatment groups (see S1 Fig). This network structure is consistent with published data in which the dominant *Firmicutes* (*Lachnospiraceae*) and *Bacteroidetes* (*Barnesiella*) are devastated by antibiotic administration [51,52]. Furthermore, the clustered structure (Fig 2D) supports the established mechanism of *C*. *difficile* colitis: loss of normal gut flora, which normally suppresses opportunistic infection (clindamycin cluster), and the presence of *C*. *difficile* at a minimum inoculum (*C*. *difficile* cluster) [10,53]. The network clusters have a single route of interaction between *Barnesiella* and *C*. *difficile*.

The negative influence of *Barnesiella* on *C*. *difficile* is in agreement with recently published findings in which *Barnesiella* was strongly correlated with *C*. *difficile* clearance [54]. The role of *Barnesiella* as an inhibitor of another pathogen (vancomycin-resistant *Enterococci* (VRE)) has been shown in mice [55], which is also visible in the network model as an indirect relationship between *Barnesiella* and *Enterococcus* (Fig 2D). Related species of *Bacteroidetes* have been shown to play vital roles in protection from *C*. *difficile* infection in mice [56]. Furthermore, the network structure shows that *Lachnospiraceae* positively interacts with *Barnesiella*, leading to an indirect suppression of *C*. *difficile*. Interestingly, the two *Lachnospiraceae* nodes and the “Other” node form a strongly connected component, suggesting a similar role in the network, particularly in promoting growth of *Barnesiella*, which directly suppresses *C*. *difficile*. In support of this finding, *Lachnospiraceae* has been shown to protect mice against *C*. *difficile* colonization [52,57]. Therefore, the structure of the network is both a parsimonious representation of the current data set, and is supported by literature evidence.

**Fig 3:**
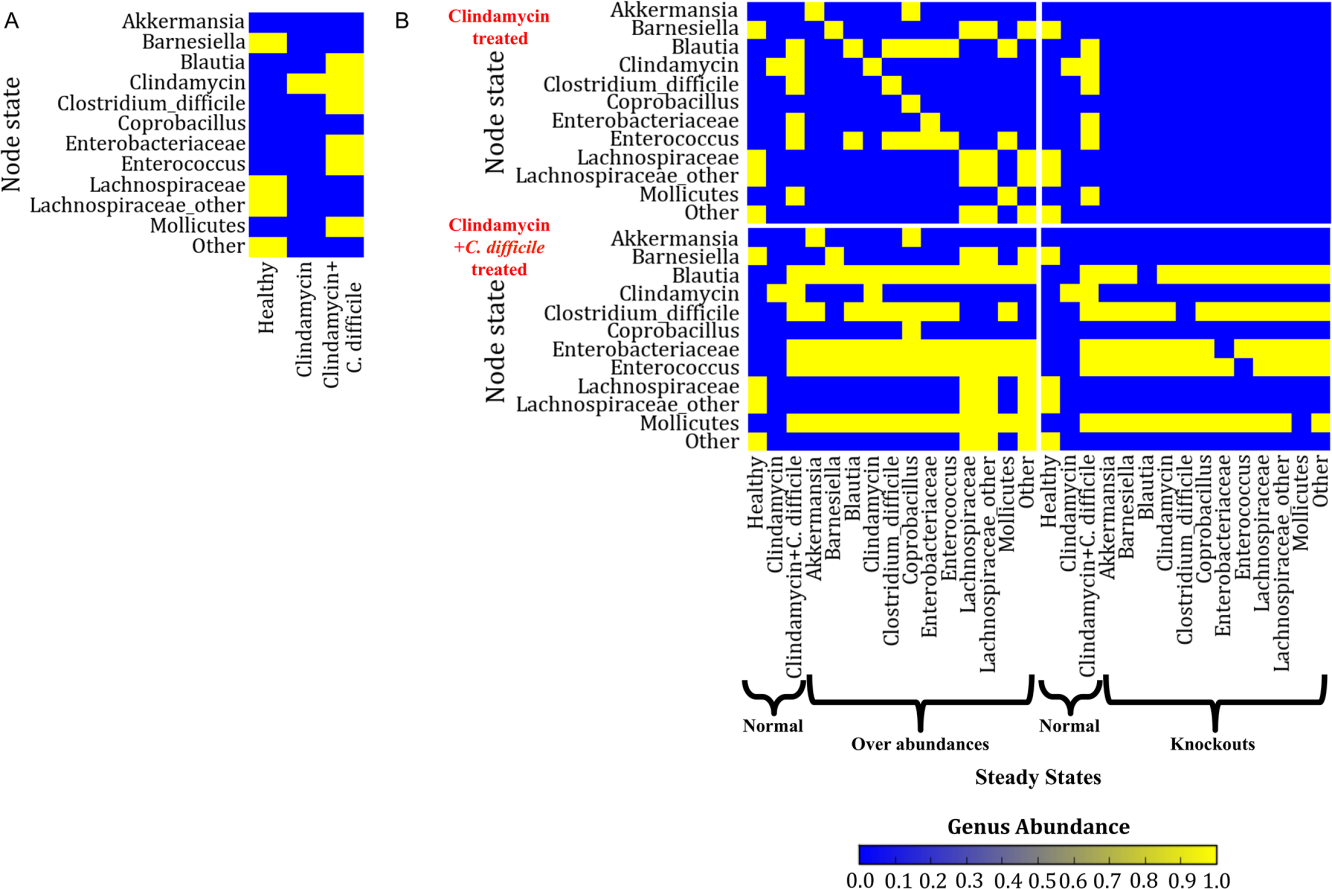
Steady states and node perturbations in the gut microbiome model. A) Heatmap of the three steady states in the gut microbiome model. These steady states are identical to steady states identified in the three experimental groups. B) The effect of node perturbations represented by four heatmaps. On the Y-axis of each of the four heatmaps are nodes (genera) in each steady state. On the x-axis of each of the four heatmaps are the steady states found under normal model conditions (i.e. no node perturbations) and also the specific perturbation of a single network node. The two heatmaps in the left column of the figure demonstrate the effect of addition (forced overabundance) of individual genera, and the two heatmaps in the right column of the figure demonstrate the effect of removal (knockout) of individual genera. The top row heatmaps show the effect of node perturbations on the clindamycin treated group and the bottom row heatmaps show the effect of node perturbations on the clindamycin+ *C*. *difficile* treatment group. *Genus abundance of 0 means present in 0% of asynchronous simulations and is indicated in blue; Genus abundance of 1 means present in all (100%) of asynchronous simulations, shown in yellow. n = 1000 simulations were applied for all Boolean model simulations.

We applied dynamic analysis using the synchronous updating scheme (see **Methods**) to determine all the possible steady states of the microbiome network model. In a 12 node network, there are 2^12^ possible network states. We employed model simulations using the synchronous updating scheme to visit all possible network states and identify all fixed points of the model. Exploration of the steady states of this network reveals 23 possible fixed point attractors (S4 Fig). Three of the identified attractors (Fig 3A) are in exact agreement with the experimentally identified terminal time points of binarized genus abundances (Fig 2C). These attractors make up a small subset of the entire microbiome network state space (S2 Table).

The attractor landscape can be divided into six groups based on abundance patterns they share (S4 Fig). Group 1 is made up of a single attractor wherein all genera are absent (OFF). The second group attractor consists of the experimentally defined healthy state (Attractor 2) and genera in the *C*. *difficile* subnetwork which can be abundant (ON) independent of the clindamycin subnetwork. The third grouping has the clindamycin treated steady state (Attractor 7) and genera in the *C*. *difficile* subnetwork that can survive in the presence of the clindamycin. Group 4 contains the clindamycin plus *C*. *difficile* steady state (Attractor 12) and its subsets in which one or both of the source nodes *Mollicutes* and *Enterobacteriaceae* are absent. Group 5 contains attractors in which clindamycin is absent and *C*. *difficile* is present. Even if clindamycin is absent, our model suggests that *C*. *difficile* can thrive if *Lachnospiraceae* and *Barnesiella* are absent, i.e. these states represent a clindamycin-independent loss of *Lachnospiraceae* and *Barnesiella*. Lastly, group 6 attractors have both clindamycin and *C*. *difficile* as OFF. *Blautia* and *Enterococcus* are always abundant in these attractors. Indeed, because of the mutual activation between *Blautia* and *Enterococcus* they always appear together. Attractors in this group may also include the abundance (ON state) of the source nodes *Mollicutes* and *Enterobacteriaceae*.

### Perturbation Analysis

We next explored the perturbation of genera in the gut microbiome network model. We considered the clinically relevant question of which perturbations might alter the microbiome steady states produced by clindamycin or clindamycin+*C*. *difficile* treatment after clindamycin treatment was removed. Thus, we considered the clindamycin-treated steady state (Attractor 7 in S3 Fig) and the clindamycin+*C*. *difficile* treated steady state (Attractor 12) as initial conditions and assumed that clindamycin treatment was stopped. Our simulations, employing asynchronous update (see **Methods**), indicate that for both initial conditions, only the state of clindamycin changes after the treatment is stopped; these steady states become Attractor 1 and Attractor 19, respectively (S4 Fig). In other words, the steady states remain identical in the absence of clindamycin. We next explored the effect of addition (overabundance; Fig 3B, left column) and removal (knockout; Fig 3B, right column) of individual genera, simultaneously with the stopping of clindamycin treatment, on the model predicted steady states. For the perturbation analysis, the model was initialized from the clindamycin treated steady state (Fig 3B, top row) or the clindamycin+*C*. *difficile* steady state (Fig 3B, bottom row). From the clindamycin treated state, addition of *Lachnospiraceae* or “Other” nodes restores the healthy steady state; however, no removal restore the healthy steady state (Fig 3B). From the clindamycin+*C*. *difficile* state, addition of *Barnesiella*, *Lachnospiraceae*, or “Other” nodes lead to a shift toward the healthy steady state (suppression of *C*. *difficile*).

### Generating Genus-Level Metabolic Reconstructions

Species-level reconstructions from the genus *Enterobacteriaceae* contained the most reactions on average (1335), while those from *Mollicutes* contained the least (485) (S3 Table). The *Barnesiella* and *Enterococcus* reconstructions contained the most unique reactions (S4 Table) and, interestingly, also displayed more overlap in reaction content between each other (503 reactions) than was observed between any other pair of reconstructions (S5 Table). *Lachnospiraceae* and *Barnesiella* had the next highest degree of overlap (424 reactions). *Mollicutes* and *Coprobacillus* had the least degree of overlap (363 reactions) (S5 Table). Note that the metabolic reconstructions produced by the SEED framework are draft quality, and as such, may lack the predictive power of well-curated metabolic reconstructions.

### Subsystem Enrichment Analysis

**Fig 4:**
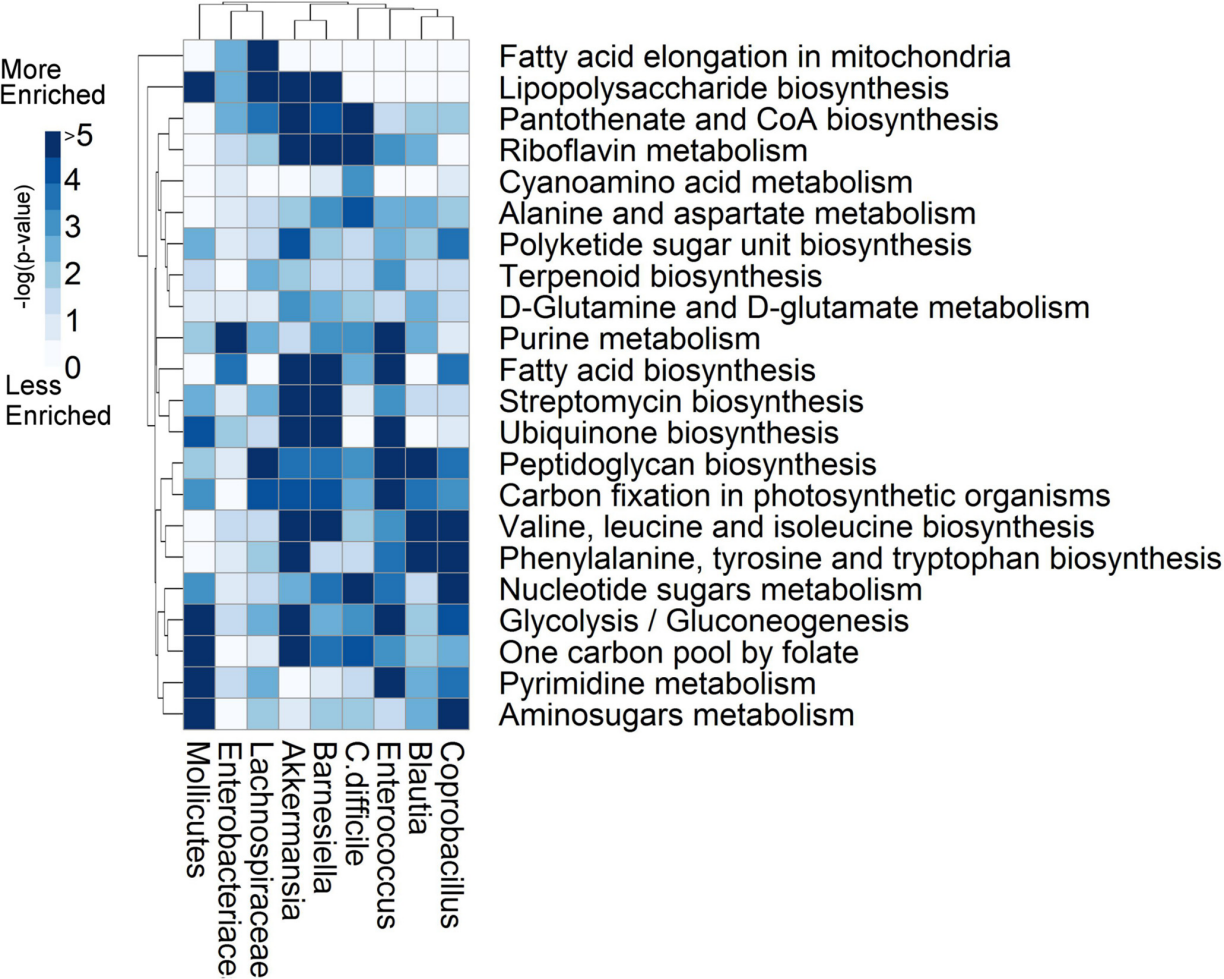
Subsystem enrichment analysis highlights metabolic differences between taxa. The p-values from the enrichment analysis are log-transformed and negated, such that darker regions indicate greater enrichment. The enrichment analysis quantifies the likelihood that a given subsystem (row) would be as highly abundant as observed within a given metabolic reconstruction (column) by chance alone. A subset of 22 interesting subsystems is shown here. Subsystems of interest include those for which all taxa are enriched, such as glycolysis, and nucleotide sugars metabolism, highlighting the fact that all taxa contain relatively full compliments of reactions within those subsystems. Similarly, subsystems for which a single genus differs from the remaining genera are interesting, such as cyanoamino acid metabolism, where *C*. *difficile* is highly enriched for reactions in that subsystem. Some subsystems are differentially enriched between *Barnesiella* and *Lachnospiraceae*, and *C*. *difficile* such as lipopolysaccharide biosynthesis and cyanoamino acid metabolism.

Enrichment analysis was performed for the 99 unique subsystem annotations that were observed in the community. 22 subsystems displayed interesting enrichment patterns with respect to the structure of the interaction network (Fig 4). The subsystems for glycolysis/gluconeogenesis and nucleotide sugars metabolism are enriched in all taxa, highlighting the fact that all taxa contain relatively full complements of reactions within those subsystems. Interestingly, *C*. *difficile* is highly enriched for reactions in cyanamino acid metabolism compared to all other genera. Lipopolysaccharide (LPS) biosynthesis and cyanoamino acid metabolism subsystems are differentially enriched between *C*. *difficile* and both *Barnesiella* and *Lachnospiraceae*. Between *Barnesiella* and *Enterococcus*, *Barnesiella* is more highly enriched for d-glutamine and d-glutamate metabolism, pantothenate and CoA biosynthesis, LPS biosynthesis. With respect to *Enterococcus*, *Barnesiella* is less highly enriched in pyrimidine metabolism, and phenylalanine, tyrosine, and tryptophan biosynthesis.

### Generating Metabolic Competition and Mutualism Scores

**Fig 5:**
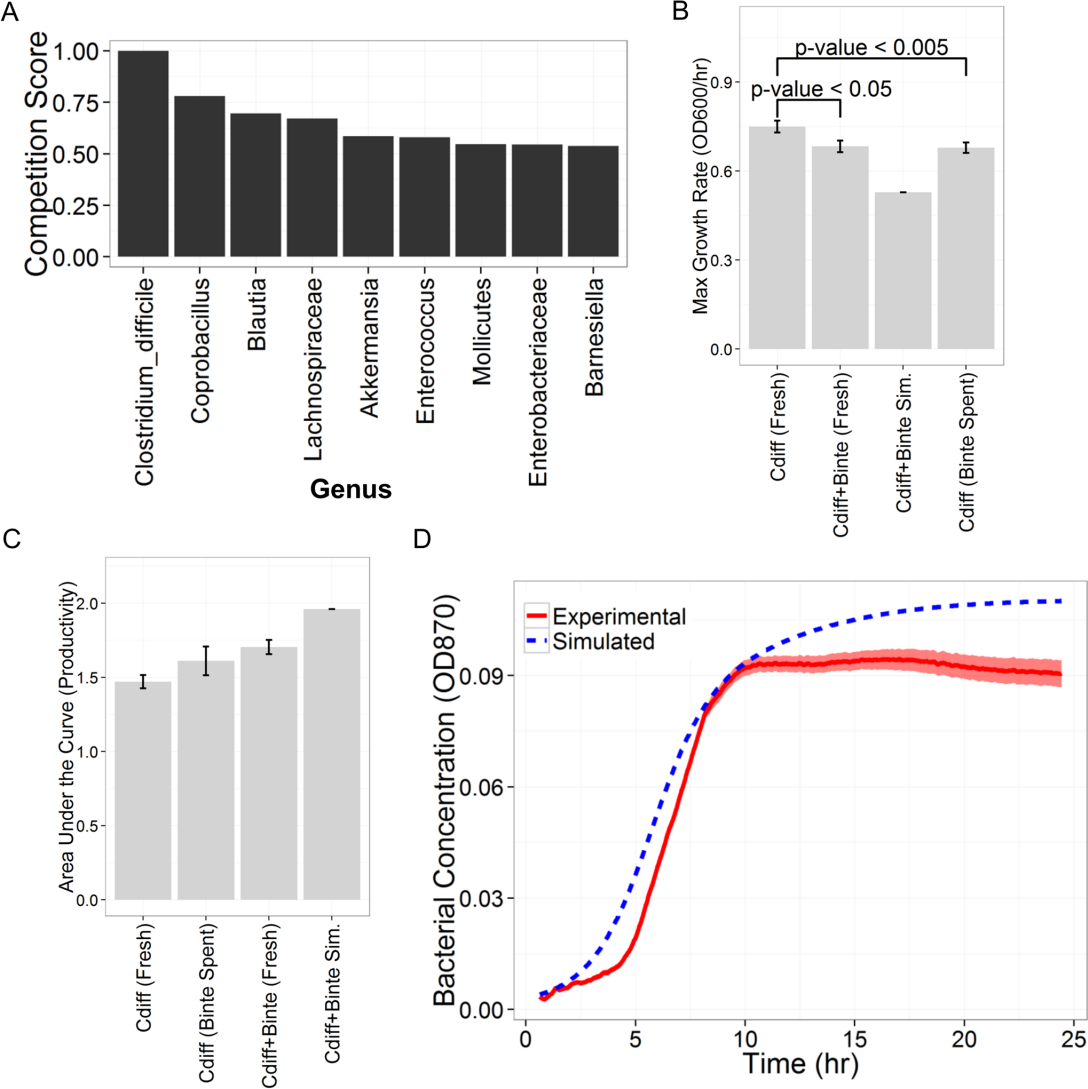
Metabolic competition scores and *in vitro* data indicate a non-metabolic interaction mechanism. A) Competition scores for all pairs of genera with *C*. *difficile*. Notice that *Barnesiella* has nearly the lowest competition score. B) Maximum growth rates for all growth conditions. *C*. *difficile* grew more slowly in *B*. *intestinihominis* spent media (n = 16, p-value < 0.005, by one-sided Wilcoxon rank sum test). The co-culture with both *B*. *intestinihominis* and *C*. *difficile* grew more slowly than *C*. *difficile* alone (n = 16, p-value < 0.05, by one-sided Wilcoxon rank sum test). C) Area under the curve (AUC) was not significantly different for *C*. *difficile* in fresh media or *B*. *intestinihominis* spent media (n = 16, p-value = 0.22 by one-sided Wilcoxon rank sum test). D) The experimental (red, solid line) and simulated (blue, dashed line) co-culture growth curves. “Binte” indicates *B*. *intestinihominis*, while “Cdiff” stands for *C*. *difficile*. On average, the experimental co-culture growth curves maintained a lower density than the simply additive null model. Error bars represent the standard error of the mean from 16 independent replicates.

The metabolic reconstructions were used to explore the potential metabolic underpinnings of the inferred interaction network. Competition scores were generated for all pairwise relationships between the genera considered in the model (self-edges were excluded). The two *Lachnospiraceae* genera were treated as metabolically identical, and the “Other” group was excluded. We grouped pairs of genera into five groups based on being connected by a positive or negative edge, a negative or positive path (meaning an indirect relationship), or no path. A positive relationship was found between competition score and edge type in the interaction network (i.e. positive edges tend to have a higher competition score), which was not statistically significant, perhaps due to the small sample size (p-value = 0.058 by one-sided Wilcoxon rank sum test) (S5A Fig). The mutualism score did not display any obvious trends with respect to the network structure (S5B Fig). All pairs with inferred edges exhibited relatively high competition scores and low mutualism scores (S5C Fig). *Barnesiella*, a key inhibitor of *C*. *difficile* in the interaction network, holds the second smallest competition score with *C*. *difficile* (see Fig 5A). *Barnesiella* and *C*. *difficile* also have the highest mutualism score among all interacting pairs in the network (S5C Fig).

The positive relationship between edge type and competition score suggests that more metabolic similarity between genera tends to foster positive interaction. The converse is also true, where less metabolic similarity tends to foster negative interactions (S5A Fig). Here, “positive/negative interaction” is derived from the Boolean model, where a positive edge between species A and B indicates that if A is ON at time t, then B is likely to turn ON at t+1.

### Co-culture and Spent Media Experiments

*Barnesiella* *intestinihominis* was chosen as a representative species for the genus Barnesiella for the in vitro experiments. *C*. *difficile* grew more slowly in *B*. *intestinihominis* spent media (n = 16, p-value < 0.005, by one-sided Wilcoxon rank sum test) (Fig 5B). The co-culture with both *B*. *intestinihominis* and *C*. *difficile* grew more slowly than *C*. *difficile* alone (n = 16, p-value < 0.05, by one-sided Wilcoxon rank sum test) (Fig 5B). *C*. *difficile* area under the growth curve (AUC), a measure of the achieved bacterial density over the experiment, was not statistically different between growth in fresh media and *B*. *intestinihominis* spent media (n = 16, p-value = 0.22 by one-sided Wilcoxon rank sum test). However, the co-culture displayed a much lower AUC than expected under a null model of interaction (in which the two species do not interact) (Fig 5C). Examining the co-culture growth curve, it maintained a consistently lower density than a null model (Fig 5D).

## Discussion

**Fig 6:**
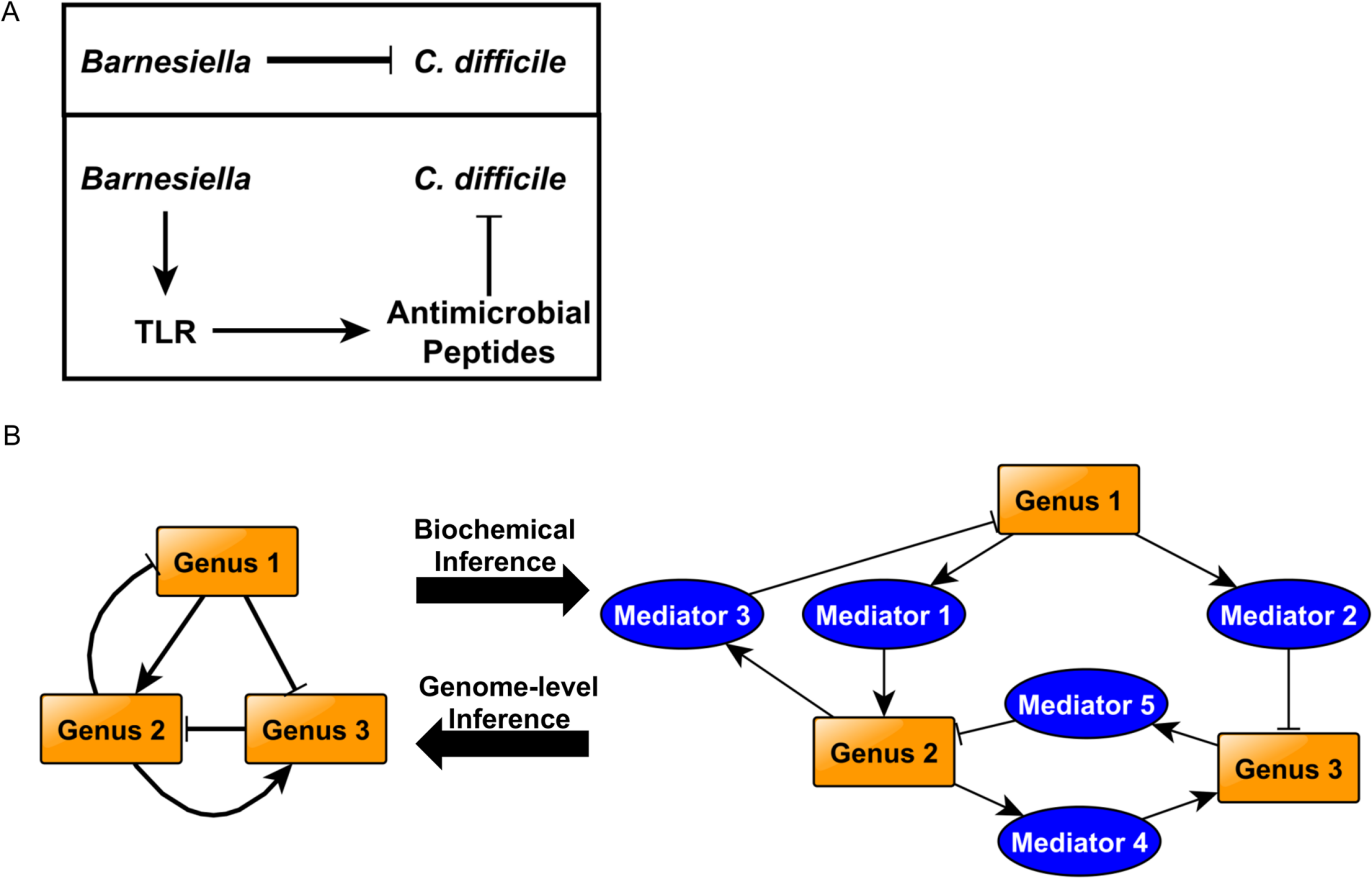
Computational models can bring us closer to true interaction networks. A) Potential inhibitory mechanisms include direct inhibition of *C*. *difficile* by *Barnesiella* (e.g. via competition for scarce resources, or toxin production), or indirect inhibition (e.g. through a host antimicrobial response). B) A great deal has been published on the topic of network inference from complex data sets, and more can be done to improve inference methods. Particularly for microbial interaction networks, it is essential to identify, not only the nature of the interactions, but also the underlying mechanisms. Metagenomic genus abundance information can be used to infer causal relationships between bacteria; however, other information sources are required to determine the exact nature of these interactions. Each individual network edge may have very different underlying causes (metabolic, physical interaction, toxin-based, etc.). Including more tools in the pipeline, such as metabolic network reconstructions, bioinformatics tools, etc., will help elucidate these mechanisms, allowing far more rapid hypothesis generation, leading to a more focused effort in the wet lab.

Here we have developed a novel strategy for generating a dynamic model of gut microbiota composition by inferring relationships from time series metagenomic data (Fig 1). To our knowledge, this is the first Boolean dynamic model of a microbial interaction network and the first Boolean model inferred from metagenomic sequence information. Metagenomic sequencing is a powerful tool that tells us the consequences of microbial interaction—changes in bacterial abundance. Bacterial interactions are, in fact, mediated by the many chemicals and metabolites the bacteria use and produce. In a network sense these relationships are a bipartite graph; bacterial genera produce chemicals/metabolites, which have an effect on other bacteria. Because there is no comprehensive source for the bacterial metabolites and their effect on other bacterial genera, we infer the effects of genera on each other from the relative abundances of genera in a set of microbiome samples, and we employ genome-scale metabolic reconstructions to gain insight into these relationships (Fig 6B). Binarization of the microbial abundances clarifies these relationships and is the starting point for the construction of a dynamic network model of the gut microbiome. Interestingly, principal component analysis demonstrates that the time series data clusters by experimental treatment group, suggesting that our initial assumption of binary relationships does not lead to significant information loss (Fig 2A and 2B).

We analyze the topological and dynamic nature of the gut microbiome, focusing on the effect of clindamycin antibiotic and *C*. *difficile* infection on gut microbial community structure. We generate a microbial interaction network and dynamic model based on time-series data from a population of mice. We validate a key edge in this interaction network between *Barnesiella* and *C*. *difficile* through an *in vitro* experiment. Consistent with the literature, our model affirms that solely inoculating a healthy microbiome with *C*. *difficile* is insufficient to disrupt the healthy intestinal tract microbiome. Additionally, our results demonstrate that clindamycin treatment has a tremendous effect on the microbiome, greatly reducing many microbial genera, and that during the time *C*. *difficile* is present, a certain subset of bacteria come to dominate the microbiome (S1 and S2 and 2C Figs).

Our dynamic network model reveals the steady state conditions attainable by this microbial system, how those steady states are reached and maintained, how they relate to the health or disease status of the mice, and how targeted changes in the network can transition the community from a disease state to a healthy state. Furthermore, we examine genome-scale metabolic network reconstructions of the taxa represented in this community, examine broad metabolic differences between the taxa in the community, and probe how metabolism could—and could not—contribute to the mechanistic underpinnings of the observed interactions.

### Network Structure

The first feature that stands out in the inferred interaction network is its clustered structure. Clindamycin has a strong influence on the subnetwork containing the two *Lachnospiraceae* nodes and *Barnesiella*. The other subnetwork contains *C*. *difficile* and other genera that become abundant during *C*. *difficile* infection (Fig 2D). Also worth noticing are the two contradicting edges in the network, between *Coprobacillus* and *Blautia*, and the self-edges for *Blautia* (Fig 2D). These arise from rules in the Boolean model that are context-dependent. Such context-dependent rules can manifest as opposite edge types, depending on the state of other nodes in the network. Context-dependent interactions have been demonstrated in many microbial pairings, and nutritional environments can even be designed to induce specific interaction types [58]. It is possible that subtle environmental changes over the course of the experiment altered conditions in a way that flipped the *Coprobacillus*-*Blautia* interaction. Because the interaction network is derived from time-series data, it is possible to estimate causality, and therefore, derive a directed graph. A directed network with clear, causative interactions can be used to study community dynamics. This is in contrast with association networks, which are often derived from independent samples, and cannot determine direction of causality [48,59–61]. Such networks are more limited in utility because they cannot be used to predict system behavior over time, or system responses to perturbations [24,62]. Note that the inferred network structure represents a set of hypotheses as to potential interactions among genera. Determining whether or not the interactions truly occur requires further experimentation, similar to the experimentation completed to validate the edge between *Barnesiella* and *C*. *difficile*.

### Experimental Validation of *Barnesiella* Inhibition of *C*. *difficile*

We experimentally validated a key edge in the interaction network, and showed that *Barnesiella* can in fact slow *C*. *difficile* growth. *C*. *difficile* was grown alone, in co-culture with *B*. *intestinihominis*, and in *B*. *intestinihominis* spentmedia. *C*. *difficile* grew more slowly in both co-culture and spent-media conditions. Though moderate, the effect was statistically significant (Fig 5B). The fact that *C*. *difficile* growth rate was inhibited under spent-media conditions indicates that *B*. *intestinihominis*-mediated inhibition does not require *B*. *intestinihominis* to “sense” the presence of *C*. *difficile*. Further, *C*. *difficile* growth on *B*. *intestinihominis* spent media demonstrates that the two species have different nutrient requirements. Whether the reduction in growth rate is a result of nutritional limitations (*e*.*g*. *C*. *difficile* resorts to a less preferred carbon source) is unknown, but unlikely given the AUC data.

The AUC—a summation of the OD over the entire time course—is a measure of the total bacterial density achieved over the course of the experiment. It can be thought of as a single metric combining growth rate and biomass production over time. Examining the AUC for all conditions showed that *C*. *difficile* AUC did not significantly change between fresh media and spent media (Fig 5C). Thus, *C*. *difficile* was able to produce comparable overall biomass despite a reduction in growth rate, further demonstrating that nutrient availability was sufficient in the spent media condition. The AUC for the co-culture was much lower than expected in a simulated null model (Fig 5C). Apparently, in co-culture, the total community biomass production capacity is reduced from what would be expected in a scenario without species interaction. Thus, there is a measurable negative interaction between *B*. *intestinihominis* and *C*. *difficile* in co-culture that impacts biomass production. This can be observed over the full time-course of the co-culture, where the overall density is consistently lower than what would be expected in a null model (Fig 5D).

### Network Dynamics and Perturbation Analysis

Computational perturbation analysis showed that forced overabundance of *Barnesiella* led to a shift from the “disease” state (clindamycin+ *C*. *difficile* treatment group) to a state highly similar to the original healthy state (loss of *C*. *difficile)*. This result is particularly interesting from a therapeutic design standpoint. In this case, the model results indicate that *Barnesiella* may serve as an effective probiotic. Model-driven analysis can be used to identify candidate organisms for probiotic treatments. Recent work by Buffie *et al*. performed a proof-of-concept study in which they used statistical models to identify candidate probiotic organisms, which were then tested on a murine model of *C*. *difficile* infection [54]. This model-driven approach can be favorably contrasted with the brute-force experimental approach in which successive combinations of microbes are tested until a curative set is found [56]. The model-driven approach requires far fewer experiments, and saves time and resources. While the computational model presented here differs from that used by Buffie *et al*., the integration of computational models in probiotic design has been shown to be a feasible, effective approach. Improved tools, such as the perturbation analysis presented here, will surely accelerate the probiotic design process and shorten the path to the clinic.

### Metabolic Competition Scores Point towards a Non-metabolic Interaction Mechanism

Genome-scale metabolic network reconstructions can be used to estimate the interactions between microbes in a complex community based purely on genome sequence data. Our use of genus-level metabolic network reconstructions (a union of several species-level reconstructions) may not reflect the unique, species-level interactions and heterogeneity within a community. This higher-level model will only capture broad trends and the possible extent of metabolic capacity within a genus. Furthermore, the draft status of these models precludes the effective application of flux balance analysis (FBA) to estimate interactions among genera. This is due to the established lack of precision in draft reconstructions in predictions of growth rates and substrate utilization patterns [63], and the sensitivity of interaction models to metabolic environment and model structure [58,64]. Future efforts to infer metabolic interactions using FBA and well-curated metabolic networks could provide deeper insights into specific metabolites that are shared (or competed for) between specific microbial pairs.

The application of competition scores demonstrated here (S5A Fig) could potentially be used to quickly establish a rough expectation (notice the spread of competition scores for the species pairs not connected by a path through the network) for community structure—based solely on genomic information—that can then be tested experimentally. Interestingly, the fact that higher competition score is associated with more positive interactions inferred from the Boolean model relates to previous work that demonstrates that higher competition scores were associated with habitat co-occurrence [46]. In this same work, the authors suggest that this effect is due to habitat filtering; that is, microbes with similar metabolic capabilities tend to thrive in similar environments. It has been shown experimentally that microorganisms from the same environment tend to lose net productivity in batch co-culture, indicating similar metabolic requirements [65]. Thus, it appears that metabolically similar organisms tend to co-locate to similar niches, and over evolutionary time, co-localized organisms tend to develop positive relationships with each other.

Understanding this relationship between competition score and interaction type leads to the conclusion that negative interactions are probably not caused by metabolic competition. Of all the genus competition scores with *C*. *difficile*, *Barnesiella* showed the second lowest (Fig 5A). In other words, *Barnesiella* is among the least likely to share a similar metabolic niche with *C*. *difficile*, which fits with the broad trend mentioned above. The fact that the competition score between *C*. *difficile* and *Barnesiella* is so low strongly suggests that the negative interaction between them is due, not to competition for scarce resources (although it does not completely exclude the possibility), but rather to some non-metabolic mechanism. The similarity in reaction content between *Barnesiella* and *Enterococcus* indicates similar network structure (S5 Table), and yet, *Enterococcus* does not inhibit *C*. *difficile* in the inferred interaction network (Fig 2D). Either the differences that are present between *Barnesiella* (65 unique reactions) and *Enterococcus* (36 unique reactions) are hints at the mechanism of interaction, or metabolism does not play a significant role in *C*. *difficile* inhibition in the environment of the gut. For example, enrichment analysis showed that that, with respect to *Enterococcus*, *Barnesiella* is more highly enriched for d-glutamine and d-glutamate metabolism, pantothenate and CoA biosynthesis and LPS biosynthesis. With respect to *Enterococcus*, *Barnesiella* is less enriched in pyrimidine metabolism, and phenylalanine, tyrosine, and tryptophan biosynthesis. The possible role of LPS is discussed further on. The possible roles of these other metabolic pathways in *C*. *difficile* inhibition is unclear.

There is experimental evidence that *Barnesiella* (and other normal flora) may combat pathogen overgrowth through non-metabolic mechanisms. As a first step, it has been shown that VRE can grow in sterile murine cecal contents—indicating the presence of sufficient nutrition to support VRE—but is inhibited in saline-treated cecal contents—indicating that live flora are needed to suppress VRE growth, and that this suppression is not through nutrient sequestration [66]. Further, the presence of *B*. *intestinihominis* has been demonstrated to prevent and cure VRE infection in mice [55], and is strongly correlated with resistance to *C*. *difficile* infection in mice [54]. Clearly, *Barnesiella* plays a key role in pathogen inhibition, and pathogen inhibition can be caused by mechanisms other than nutrient competition.

This non-metabolic mechanism may be direct or indirect (Fig 6A). We demonstrated *in vitro* that *B*. *intestinihominis* can inhibit *C*. *difficile* growth rate (Fig 5C and 5D). The fact that *C*. *difficile* grows on *B*. *intestinihominis* spent media at all indicates that the metabolic requirements of the two species are different, which is consistent with our computational results supporting the hypothesis that *C*. *difficile* and *Barnesiella* do not compete metabolically (Fig 5B). Further, *C*. *difficile* is moderately inhibited both in co-culture with *B*. *intestinihominis* and in *B*. *intestinihominis*-spent media, indicating a direct mechanism of inhibition. In further support of a direct mechanism, it has been shown that *Clostridium scindens* inhibits growth of *C*. *difficile* through the production of secondary bile acids [54]. Perhaps *Barnesiella* works through an analogous mechanism *in vivo*, enhancing the moderate inhibition observed *in vitro*.

In support of an additional indirect mechanism of bacterial interaction, Buffie and Pamer, in a recent review, hypothesized that the normal flora (of which *Barnesiella* is a member) may prevent pathogen overgrowth by stimulation of a host antimicrobial response [67] (Fig 6A). Specifically, they point out that *Barnesiella* can activate host toll-like receptor TLR signaling, which activates host antimicrobial peptide production. For example, LPS and flagellin have been shown to stimulate the host innate immune response through toll-like receptor (TLR) signaling and production of bactericidal lectins [68,69]. *Barnesiella* shows enrichment for LPS biosynthesis pathways (Fig 4). However, this mechanism did not seem to be responsible for inhibition of VRE by *Barnesiella* [55]. An indirect, host-mediated mechanism is further supported by the fact that members of the normal gut flora can interact differently with pathogens depending on the host organism [54]. Regardless, any indirect mechanism is in addition to the direct inhibitory mechanism observed *in vitro*. Both direct and indirect mechanisms may play a role *in vivo*, and further work is needed to clearly discern the underlying process that allows *Barnesiella* to play this protective role.

We demonstrate that dynamic Boolean models capture key microbial interactions and dynamics from time-series abundance data in a murine microbiome. We show that this computational approach enables exhaustive *in silico* perturbation, which leads to fast candidate selection for probiotic design. We further describe the use of genome-scale metabolic network reconstructions to explore the metabolic potential attributed to community members, and to estimate metabolic competition and cooperation between members of the microbiome community. Analysis of genome-scale metabolic network reconstructions indicates that *Barnesiella* likely inhibits *C*. *difficile* through some non-metabolic mechanism. We present empirical *in vitro* evidence that *B*. *intestinihominis* does in fact inhibit *C*. *difficile* growth, likely by a non-metabolic mechanism, and our findings are in good agreement with published results. We present this work as a demonstration of the use of dynamic Boolean models and genome-scale metabolic reconstructions to explore the structure, dynamics, and mechanistic underpinnings of complex microbial communities.

## Supporting Information

S1 Fig Bacterial genera abundances over time in response to clindamycin treatment and/or *C*. *difficile* inoculation. A) Genera abundance information for the nine samples. The “Healthy” population received spores of *C*. *difficile* (at t = 0 days) and did not undergo observable microbial changes, Population 2 received a single dose of clindamycin (at t = -1 days), and Population 3 received a single dose of clindamycin (at t = -1 days) and, on the following day, was inoculated with *C*. *difficile* spores (at t = 0 days). Genus abundances were measured at 0, 2, 3, 4, 5, 6, 7, 9, 12, 13, 16, and 23 days; however, not all samples had measurements at all the time points. B) Cubic spline interpolation of data points was performed such that all the same time point measurements of bacterial abundance occurred in all samples and that single day intervals were present in all datasets.
(TIF)

S2 Fig Averaged binarized genera abundances using iterative k-means binarization. Iterative k-means binarization was completed on all the samples 1000 times and average binarization is shown for each genus at each time point in each of the nine samples. If a node (genus) is binarized as 0 (OFF) at a time step, then it is colored blue, and if a node (genus) is binarized as 1 (ON) at a time step, then it is colored yellow. This figure represents the average of 1000 replicates of IKM binarization. Intermediate cell colors represent cases where a genus abundance at a time point was binarized to 1 (ON) in a fraction of the replicates.
(TIFF)

S3 Fig Averaged binarized genera abundances using iterative k-means binarization were rounded to the most probable binarized state. The most probable binarized state of each genus at each time point. If the average genus abundance binarization (S2 Fig) was greater than 0.5 (ON in over 500 of 1000 replicates), then that genus abundance was assumed to be 1 (ON) for downstream analysis. If the average genus abundance binarization was less than 0.5 (ON in less than 500 of 1000 replicates) then that genus abundance was assumed to be 0 (OFF) for downstream analysis.
(TIFF)

S4 Fig All possible steady states of the Boolean model of the gut microbiome. There are 23 predicted steady states in the Boolean model of the gut microbiome. Each attractor is a column in the heatmap and is made up of the state of each genus in the network model (rows). Each genus can be present above an activity threshold (yellow; ON) or below an activity threshold (blue; OFF). The steady states in the model are grouped based on their similarities to other steady states in the same group. The first steady state of group 2 (Attractor 2) is the healthy steady state, the first steady state of group 3 (Attractor 7) is the clindamycin treated steady state, and the first steady state of group 4 (Attractor 12) is the clindamycin + *C*. *difficile* steady state. These three steady states are directly corroborated by experimental metagenomic data.
(TIFF)

S5 Fig Competition and mutualism scores by edge and path type in Boolean network. A) Competition score values for all classes of paths through the network, including direct edges, directed paths, and no directed path. A positive relationship was found between competition score and direct edge type in the dynamic network (self-edges were excluded), which was not statistically significant, perhaps due to the small sample size (p-value = 0.058 by one-sided Wilcoxon rank sum test), but is worthy of note. B) Mutualism score values for all classes of paths through the network, including direct edges, directed paths, and no directed path. C) Competition and mutualism score plot for the interaction edges in the network. All the interactions reflect moderate to high competition scores and relatively low mutualism scores. All the interactions have a higher competition score than mutualism score. The two negative interactions (red circles) do not have higher competition scores, nor lower mutualism scores, than the positive interactions. In fact, the negative interaction between *Barnesiella* and *C*. *difficile* corresponds to the highest mutualism score.
(TIF)

S1 Table Boolean update rules for the gut microbiome network. The ruleset inferred from metagenomic sequencing information using Boolnet.
(DOCX)

S2 Table Basin size as % of total state space (unique basin size) for experimentally realized network attractors. (DOCX)

S3 Table Genus-level genome-scale metabolic network reconstruction characteristics. In this table we characterize the genus-level metabolic network reconstructions. Average model size refers to the average number of reactions in the component species reconstructions within each genus. *Akkermansia* is represented by a single species-level reconstruction, while several genera are represented by 10 species-level reconstructions. The average network overlap within a genus refers to the average number of shared reactions between any two pairs of species within the genus. Similarly, the average fraction of unique reactions refers to the average subset of reactions in a given species that are unique within the genus.
(DOCX)

S4 Table Unique reactions within genera. The genus in each row has n reactions that the genus in the columns do not have. For example, the genus-level reconstruction for *Barnesiella* contains 167 reactions that the reconstruction for *C*. *difficile* does not. Conversely, the reconstruction for *C*. *difficile* only contains 30 unique reactions that the reconstruction for *Blautia* does not already contain.
(DOCX)

S5 Table Reaction overlap between genera. The upper portion of the table contains the number of shared reaction content between all genus-level metabolic network reconstructions.
(DOCX)
