# Supplementary figures and images for: Inference of Network Dynamics and Metabolic Interactions in the Gut Microbiome
Source: PLoS Comput Biol. 2015 Jun 23;11(6):e1004338. doi: 10.1371/journal.pcbi.1004338 (PMC4478025; doi:10.1371/journal.pcbi.1004338)

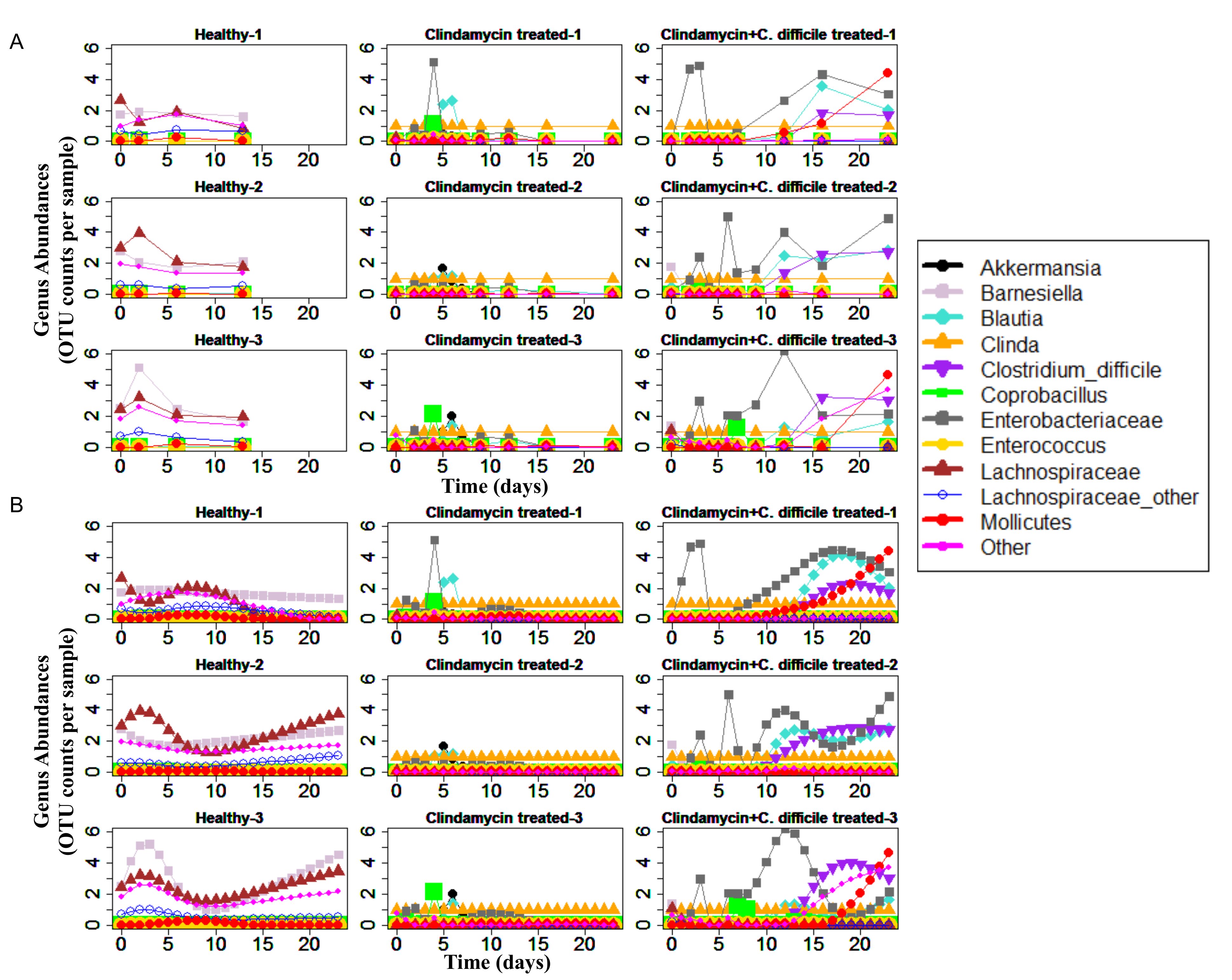

Supplement: S1 Fig — A) Genera abundance information for the nine samples. The “Healthy” population received spores of C. difficile (at t = 0 days) and did not undergo observable microbial changes, Population 2 received a single dose of clindamycin (at t = -1 days), and Population 3 received a single dose of clindamycin (at t = -1 days) and, on the following day, was inoculated with C. difficile spores (at t = 0 days). Genus abundances were measured at 0, 2, 3, 4, 5, 6, 7, 9, 12, 13, 16, and 23 days; however, not all samples had measurements at all the time points. B) Cubic spline interpolation of data points was performed such that all the same time point measurements of bacterial abundance occurred in all samples and that single day intervals were present in all datasets. (TIF) [file pcbi.1004338.s001.tif]

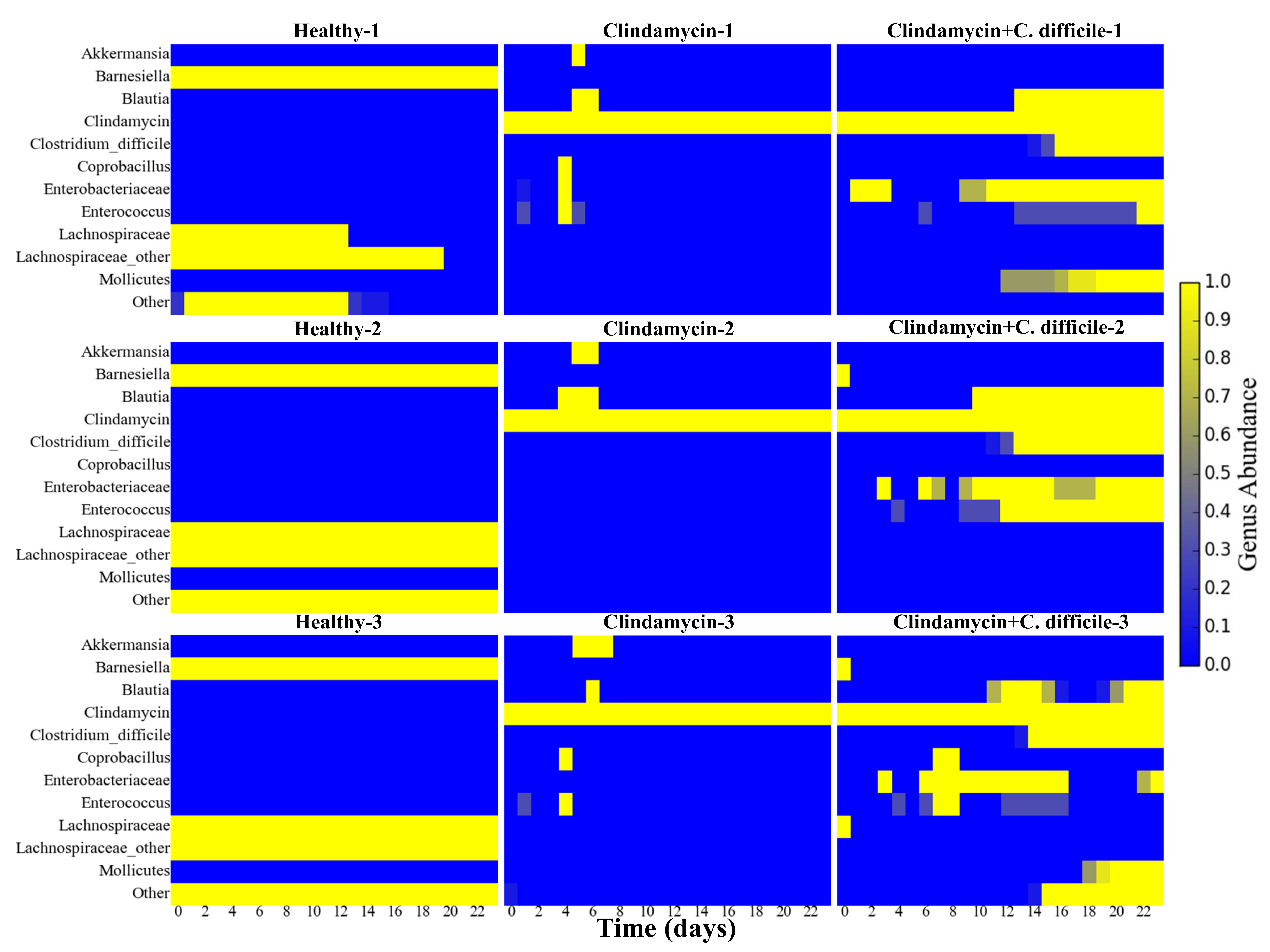

Supplement: S2 Fig — Iterative k-means binarization was completed on all the samples 1000 times and average binarization is shown for each genus at each time point in each of the nine samples. If a node (genus) is binarized as 0 (OFF) at a time step, then it is colored blue, and if a node (genus) is binarized as 1 (ON) at a time step, then it is colored yellow. This figure represents the average of 1000 replicates of IKM binarization. Intermediate cell colors represent cases where a genus abundance at a time point was binarized to 1 (ON) in a fraction of the replicates. (TIFF) [file pcbi.1004338.s002.tiff]

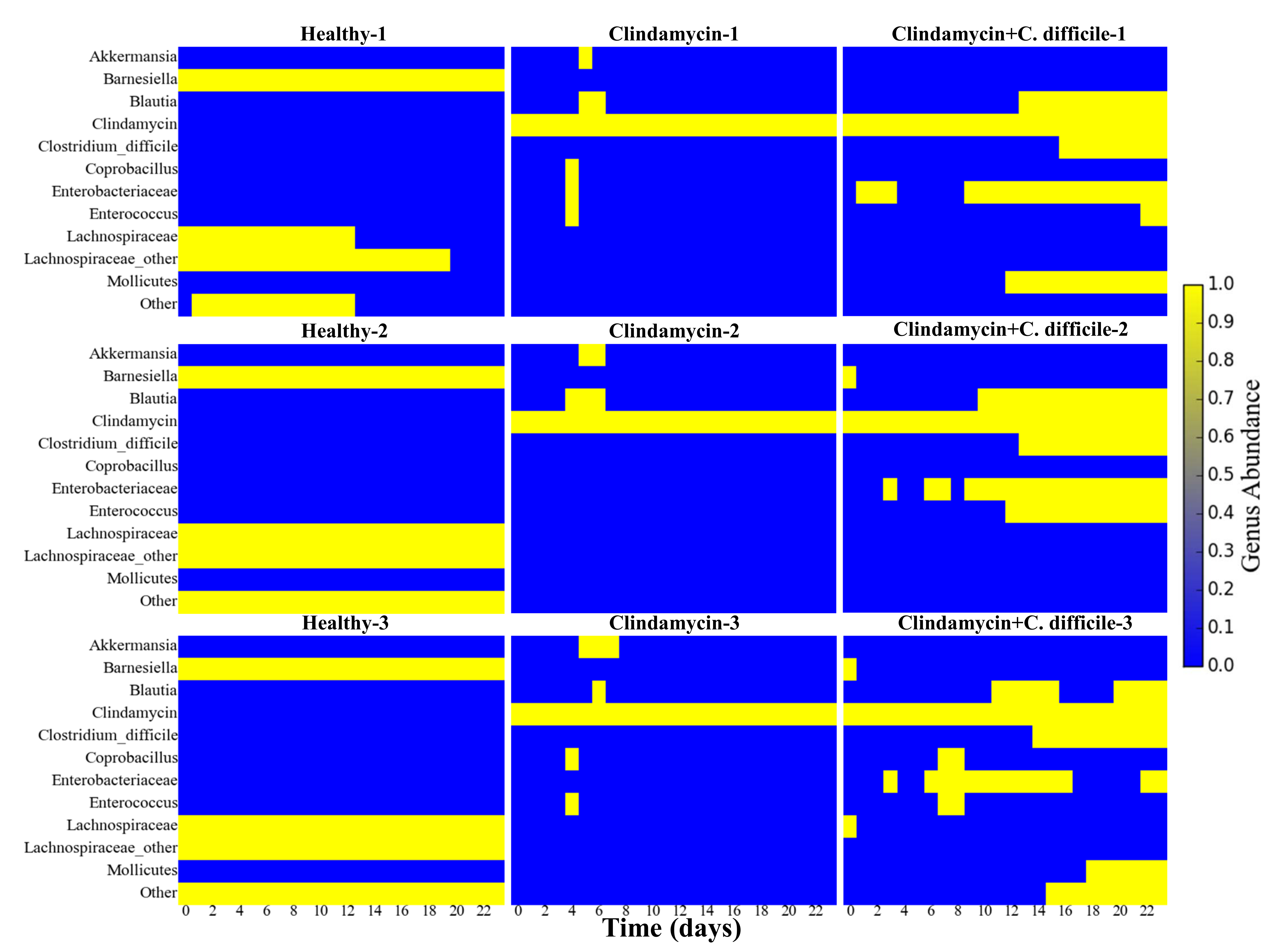

Supplement: S3 Fig — The most probable binarized state of each genus at each time point. If the average genus abundance binarization (S2 Fig) was greater than 0.5 (ON in over 500 of 1000 replicates), then that genus abundance was assumed to be 1 (ON) for downstream analysis. If the average genus abundance binarization was less than 0.5 (ON in less than 500 of 1000 replicates) then that genus abundance was assumed to be 0 (OFF) for downstream analysis. (TIFF) [file pcbi.1004338.s003.tiff]

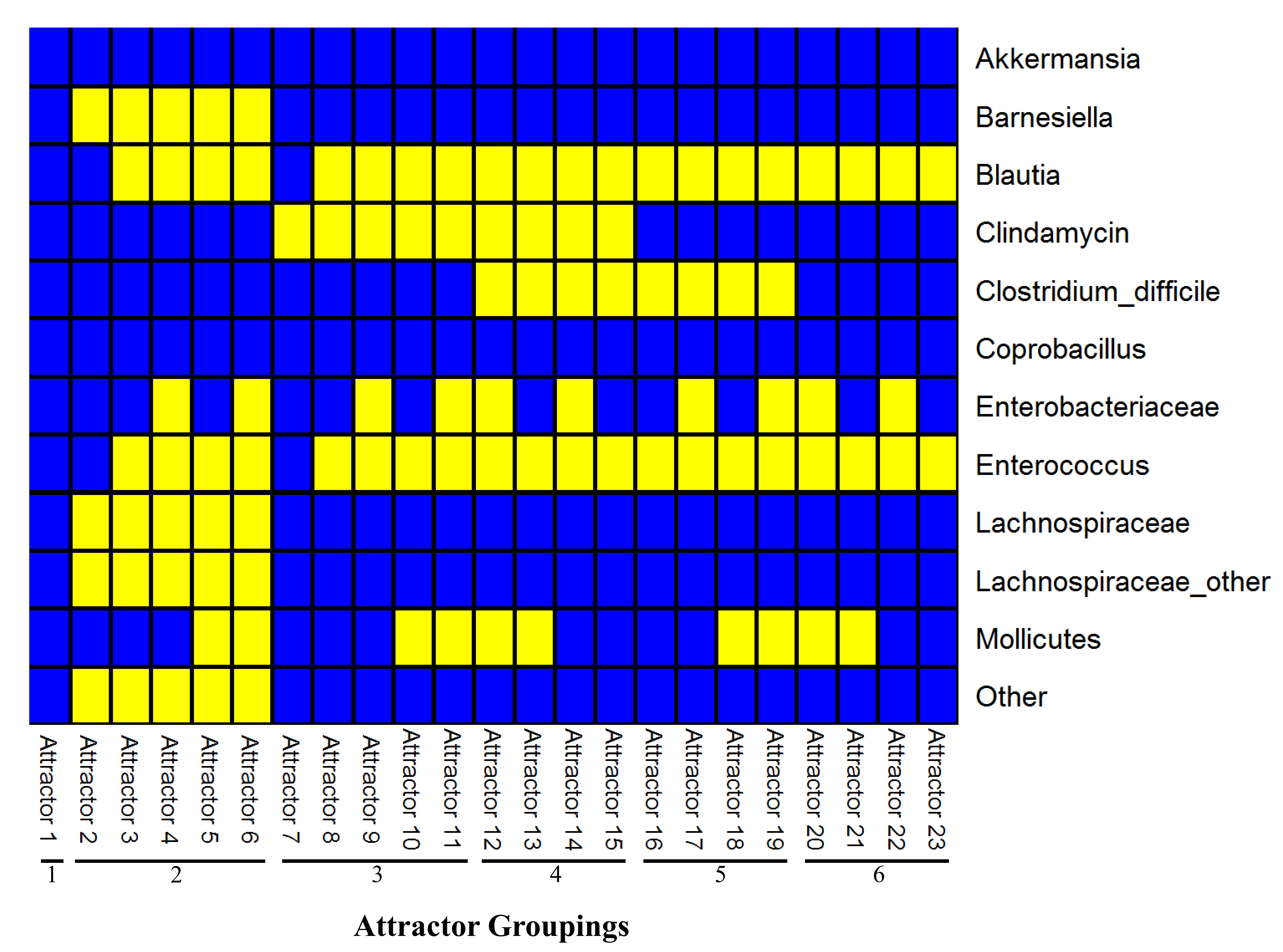

Supplement: S4 Fig — There are 23 predicted steady states in the Boolean model of the gut microbiome. Each attractor is a column in the heatmap and is made up of the state of each genus in the network model (rows). Each genus can be present above an activity threshold (yellow; ON) or below an activity threshold (blue; OFF). The steady states in the model are grouped based on their similarities to other steady states in the same group. The first steady state of group 2 (Attractor 2) is the healthy steady state, the first steady state of group 3 (Attractor 7) is the clindamycin treated steady state, and the first steady state of group 4 (Attractor 12) is the clindamycin + C. difficile steady state. These three steady states are directly corroborated by experimental metagenomic data. (TIFF) [file pcbi.1004338.s004.tiff]

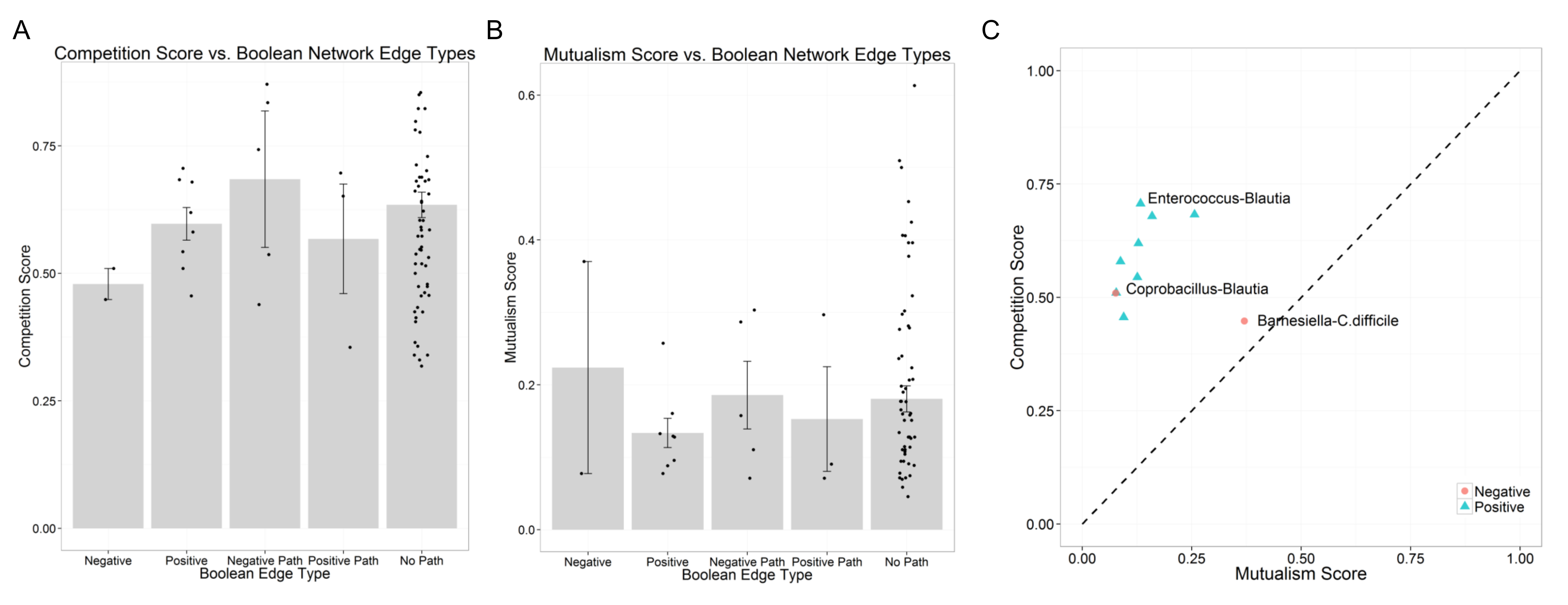

Supplement: S5 Fig — A) Competition score values for all classes of paths through the network, including direct edges, directed paths, and no directed path. A positive relationship was found between competition score and direct edge type in the dynamic network (self-edges were excluded), which was not statistically significant, perhaps due to the small sample size (p-value = 0.058 by one-sided Wilcoxon rank sum test), but is worthy of note. B) Mutualism score values for all classes of paths through the network, including direct edges, directed paths, and no directed path. C) Competition and mutualism score plot for the interaction edges in the network. All the interactions reflect moderate to high competition scores and relatively low mutualism scores. All the interactions have a higher competition score than mutualism score. The two negative interactions (red circles) do not have higher competition scores, nor lower mutualism scores, than the positive interactions. In fact, the negative interaction between Barnesiella and C. difficile corresponds to the highest mutualism score. (TIF) [file pcbi.1004338.s005.tif]
