# Supplementary material for: Inference of Network Dynamics and Metabolic Interactions in the Gut Microbiome
Source: PLoS Comput Biol. 2015 Jun 23;11(6):e1004338. doi: 10.1371/journal.pcbi.1004338 (PMC4478025; doi:10.1371/journal.pcbi.1004338)
Supplement: S1 Table — The ruleset inferred from metagenomic sequencing information using Boolnet. (DOCX) [file pcbi.1004338.s006.docx]

| **Supplemental Table 1. Boolean update rules for the gut microbiome network** | |
| --- | --- |
| **Node** | **Boolean Update Rule** |
| Akkermansia | Coprobacillus |
| Barnesiella | (Other or Lachnospiraceae_other or Lachnospiraceae) and not Clindamycin |
| Blautia | (Enterococcus or Blautia) or [(not Blautia and Coprobacillus) or (Blautia and not Coprobacillus)] |
| Clindamycin | Clindamycin |
| Clostridium_difficile | Clostridium_difficile and not Barnesiella |
| Coprobacillus | OFF |
| Enterobacteriaceae | Enterobacteriaceae |
| Enterococcus | Mollicutes or Enterobacteriaceae or not Coprobacillus or Clostridium_difficile or Blautia |
| Lachnospiraceae | (Other or Lachnospiraceae_other or Lachnospiraceae) and not Clindamycin |
| Lachnospiraceae_other | (Other or Lachnospiraceae_other or Lachnospiraceae) and not Clindamycin |
| Mollicutes | Mollicutes |
| Other | (Other or Lachnospiraceae_other or Lachnospiraceae) and not Clindamycin |
