# Supplementary material for: Inference of Network Dynamics and Metabolic Interactions in the Gut Microbiome
Source: PLoS Comput Biol. 2015 Jun 23;11(6):e1004338. doi: 10.1371/journal.pcbi.1004338 (PMC4478025; doi:10.1371/journal.pcbi.1004338)
Supplement: S2 Table — (DOCX) [file pcbi.1004338.s007.docx]

| **Supplemental Table 2. Basin size as % of total state space (unique basin size) for experimentally realized network attractors** | | |
| --- | --- | --- |
| **Attractor** | **Synchronous** | **Asynchronous** |
| Healthy | 1.563 | 4.688 (1.563) |
| Clindamycin treated | 0.147 | 0.439 (0.098) |
| Clindamycin+*C. Difficile* | 1.367 | 0.293 (0.098) |
