# Supplementary material for: Inference of Network Dynamics and Metabolic Interactions in the Gut Microbiome
Source: PLoS Comput Biol. 2015 Jun 23;11(6):e1004338. doi: 10.1371/journal.pcbi.1004338 (PMC4478025; doi:10.1371/journal.pcbi.1004338)
Supplement: S3 Table — In this table we characterize the genus-level metabolic network reconstructions. Average model size refers to the average number of reactions in the component species reconstructions within each genus. Akkermansia is represented by a single species-level reconstruction, while several genera are represented by 10 species-level reconstructions. The average network overlap within a genus refers to the average number of shared reactions between any two pairs of species within the genus. Similarly, the average fraction of unique reactions refers to the average subset of reactions in a given species that are unique within the genus. (DOCX) [file pcbi.1004338.s008.docx]

| **Supplemental Table 3. Genus level genome scale metabolic network reconstructions** | | | | |
| --- | --- | --- | --- | --- |
| **Genus** | **Average Model Size**  (average of number of reactions in member-species reconstructions) | **Number of species-level models included in each genus** | **Average Network Overlap Within genus**  (average number of shared reactions, normalized by average network size) | **Average Fraction of Unique Reactions**  (what fraction of a given species’ network is unique within its genus, normalized by average network size) |
| Akkermansia | 818 | 1 | 1 | 1 |
| Barnesiella | 898 | 2 | 0.9621 | 0.0379 |
| Blautia | 980.9 | 10 | 0.8751 | 0.0093 |
| Clostridium_difficile | 999.5 | 2 | 0.9735 | 0.0265 |
| Coprobacillus | 910.3 | 3 | 0.9821 | 0.0092 |
| Enterobacteriaceae | 1335.5 | 10 | 0.8738 | 0.0054 |
| Enterococcus | 916.4 | 10 | 0.8865 | 0.0143 |
| Lachnospiraceae | 966 | 10 | 0.85 | 0.0175 |
| Mollicutes | 485.2 | 10 | 0.8233 | 0.0268 |
