# Supplementary material for: Inference of Network Dynamics and Metabolic Interactions in the Gut Microbiome
Source: PLoS Comput Biol. 2015 Jun 23;11(6):e1004338. doi: 10.1371/journal.pcbi.1004338 (PMC4478025; doi:10.1371/journal.pcbi.1004338)
Supplement: S4 Table — The genus in each row has n reactions that the genus in the columns do not have. For example, the genus-level reconstruction for Barnesiella contains 167 reactions that the reconstruction for C. difficile does not. Conversely, the reconstruction for C. difficile only contains 30 unique reactions that the reconstruction for Blautia does not already contain. (DOCX) [file pcbi.1004338.s009.docx]

| **Supplemental Table 4. Unique reactions within genera. Each row has n reactions that column does not have.** | | | | | | | | | |
| --- | --- | --- | --- | --- | --- | --- | --- | --- | --- |
|  | **Akkermansia** | **Barnesiella** | **Blautia** | **Clostridium_difficile** | **Coprobacillus** | **Enterobacteriaceae** | **Enterococcus** | **Lachnospiraceae** | **Mollicutes** |
| Akkermansia | 0 | 80 | 80 | 95 | 86 | 66 | 76 | 103 | 113 |
| Barnesiella | 163 | 0 | 155 | 167 | 167 | 148 | 65 | 144 | 155 |
| Blautia | 65 | 57 | 0 | 48 | 85 | 65 | 52 | 61 | 92 |
| Clostridium_difficile | 62 | 51 | 30 | 0 | 70 | 53 | 45 | 40 | 84 |
| Coprobacillus | 65 | 63 | 79 | 82 | 0 | 49 | 71 | 71 | 101 |
| Enterobacteriaceae | 73 | 72 | 87 | 93 | 77 | 0 | 74 | 95 | 109 |
| Enterococcus | 130 | 36 | 121 | 132 | 146 | 121 | 0 | 134 | 153 |
| Lachnospiraceae | 90 | 48 | 63 | 60 | 79 | 75 | 67 | 0 | 80 |
| Mollicutes | 90 | 49 | 84 | 94 | 99 | 79 | 76 | 70 | 0 |
