# Supplementary material for: Inference of Network Dynamics and Metabolic Interactions in the Gut Microbiome
Source: PLoS Comput Biol. 2015 Jun 23;11(6):e1004338. doi: 10.1371/journal.pcbi.1004338 (PMC4478025; doi:10.1371/journal.pcbi.1004338)
Supplement: S5 Table — The upper portion of the table contains the number of shared reaction content between all genus-level metabolic network reconstructions. (DOCX) [file pcbi.1004338.s010.docx]

| **Supplemental Table 5. Reaction overlap between genera** | | | | | | | | | |
| --- | --- | --- | --- | --- | --- | --- | --- | --- | --- |
|  | **Akkermansia** | **Barnesiella** | **Blautia** | **Clostridium_difficile** | **Coprobacillus** | **Enterobacteriaceae** | **Enterococcus** | **Lachnospiraceae** | **Mollicutes** |
| Akkermansia | 0 | 405 | 405 | 390 | 399 | 419 | 409 | 382 | 372 |
| Barnesiella | 0 | 0 | 413 | 401 | 401 | 420 | 503 | 424 | 413 |
| Blautia | 0 | 0 | 0 | 422 | 385 | 405 | 418 | 409 | 378 |
| Clostridium_difficile | 0 | 0 | 0 | 0 | 382 | 399 | 407 | 412 | 368 |
| Coprobacillus | 0 | 0 | 0 | 0 | 0 | 415 | 393 | 393 | 363 |
| Enterobacteriaceae | 0 | 0 | 0 | 0 | 0 | 0 | 418 | 397 | 383 |
| Enterococcus | 0 | 0 | 0 | 0 | 0 | 0 | 0 | 405 | 386 |
| Lachnospiraceae | 0 | 0 | 0 | 0 | 0 | 0 | 0 | 0 | 392 |
| Mollicutes | 0 | 0 | 0 | 0 | 0 | 0 | 0 | 0 | 0 |
